# The production of the chemokine CCL2 by corneal sensory neurons initiates anti-viral immunity at the cornea and trigeminal ganglion

**DOI:** 10.1016/j.celrep.2025.116693

**Published:** 2025-12-16

**Authors:** Hongmin Yun, Kaveh Moghbeli, Peter Habib Gerges, Rylee N. Cisney, Masaaki Yoshida, William F. Hawse, William A. MacDonald, Shamsuddin A. Bhuiyan, William Renthal, Christopher J. Sullivan, Jishnu Das, Daniel H. Kaplan, Harinder Singh, Brian M. Davis, Anthony J. St. Leger

**Affiliations:** 1 School of Medicine, Department of Ophthalmology, University of Pittsburgh, Pittsburgh, PA, USA; 2 Center for Systems Immunology and Department of Immunology, University of Pittsburgh, Pittsburgh, PA, USA; 3 Department of Microbiology and Immunology, University of Pittsburgh, Pittsburgh, PA, USA; 4 Department of Pediatrics, University of Pittsburgh, Pittsburgh, PA, USA; 5 Department of Neurology, Brigham and Women’s Hospital and Harvard Medical School, Boston, MA 02115, USA; 6 Department of Neurobiology, University of Pittsburgh, Pittsburgh, PA, USA; 7 Department of Dermatology, University of Pittsburgh, Pittsburgh, PA, USA; 8 These authors contributed equally; 9 Lead contact

## Abstract

The cornea is an epithelial tissue densely innervated by sensory neurons but devoid of autonomic innervation, lymphatics, and vasculature. The simplicity of the cellular composition suggests that corneal afferents participate in tissue homeostasis by regulating immune cells. Transcriptomic analysis of retrogradely labeled corneal afferents in the trigeminal ganglion (TG) found that they express many immune-relatedgenes.Optogeneticactivation of corneal afferents increased neutrophils and monocytes in both the cornea and TG, as well as inducing phenotypic changes in natural killer (NK) cells. Unsupervised pathway analysis indicated neuronally expressed *Ccl2* as a modulator of immune cell responses. Selective deletion of neuronal *Ccl2* decreased the number of myeloid cells in the cornea and TG in response to herpes simplex virus (HSV) infection, resulting in compromised viral clearance during primary infection. These experiments demonstrate that corneal afferent activation is sufficient to trigger inflammatory responses that can assist the host in initiating anti-viral immunity.

## In brief

Yun et al. combined neuronal labeling, single-cell transcriptomics, and computational analyses to map communication between corneal neurons and immune cells. They identified neuron-derived CCL2 as recruiting immune cells during early HSV-1 infection, and loss of CCL2 reduced immune recruitment, impairing early viral control.

## Graphical Abstract

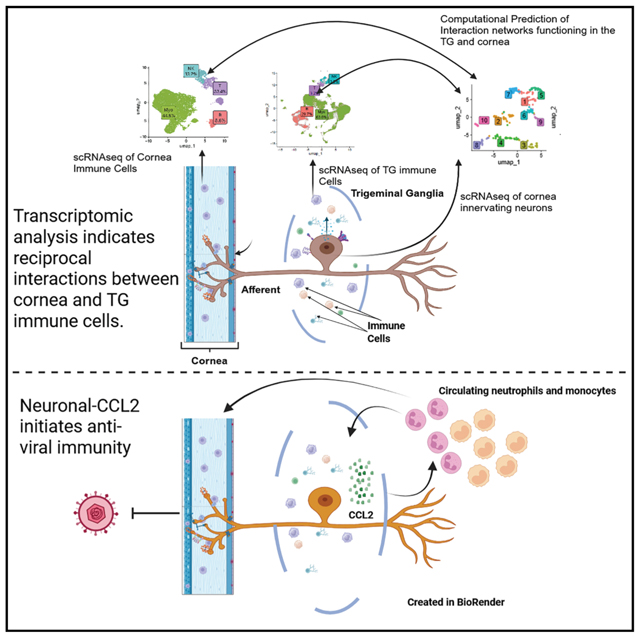

## INTRODUCTION

Recent studies suggest that sensory neurons do more than passively respond to environmental cues by eliciting reflexes. The complexity of their potential contribution to homeostasis is suggested by their heterogeneity, both with respect to function and chemical phenotype. Sensory neurons can be separated into broad classes (e.g., peptidergic or non-peptidergic), based on the expression of neuropeptides (e.g., CGRP [calcitonin gene-related peptide] and/or SP [substance P]) or their absence. Peptidergic sensory neurons have been shown to play a central role in immunosuppression in disease models as diverse as migraine and cancer,^1–3^ as well as driving type 17 inflammation in skin.^4^ In contrast, non-peptidergic neurons (i.e., those lacking peptides) have been shown to suppress mast cells via glutamate release.^5^ Moreover, sensory neurons express numerous gene products like cytokines, chemokines, and/or membrane-bound proteins (e.g., PDL1^6,7^ and neuropilin 1/2^6,8^) for the purpose of communicating with immune cells.

Corneal afferent cell bodies are located in the trigeminal ganglion (TG). They have been extensively studied, and much is known about their physiologic response properties^9–12^ and protein expression (based on immunohistochemical analysis^13–16^). Previous studies have phenotyped sensory neurons in the TG on the transcriptomic level without determining the specific tissue targets of the approximately 20,000 neurons in the mouse TG.^17–19^ As a first step in exploring potential neuroimmune interactions in the cornea, we back-labeled corneal afferents, dissociated the TG, and handpicked labeled cells for single-cell RNA sequencing (scRNA-seq) analysis. Notably, labeled neurons robustly expressed genes that are canonical markers for immune cells. To directly test whether corneal afferents could initiate immune responses, we activated corneal afferents optogenetically or via exposure to herpes simplex virus 1 (HSV-1). Briefly, optogenetic activation was sufficient to alter the corneal and TG immune cell landscape, similar to what is seen in acute stages of inflammation. Finally, the immune response to viral infection (HSV-1) was decreased in both the cornea and TG when the *Ccl2* chemokine gene was deleted selectively in peptidergic corneal afferents. These results demonstrate that corneal afferents play a significant role in corneal immune homeostasis and are positioned to respond rapidly to changes in the corneal environment.

## RESULTS

### Assessing the identities of cornea-innervating afferents at the periphery and in the TG

**Figure 1.**
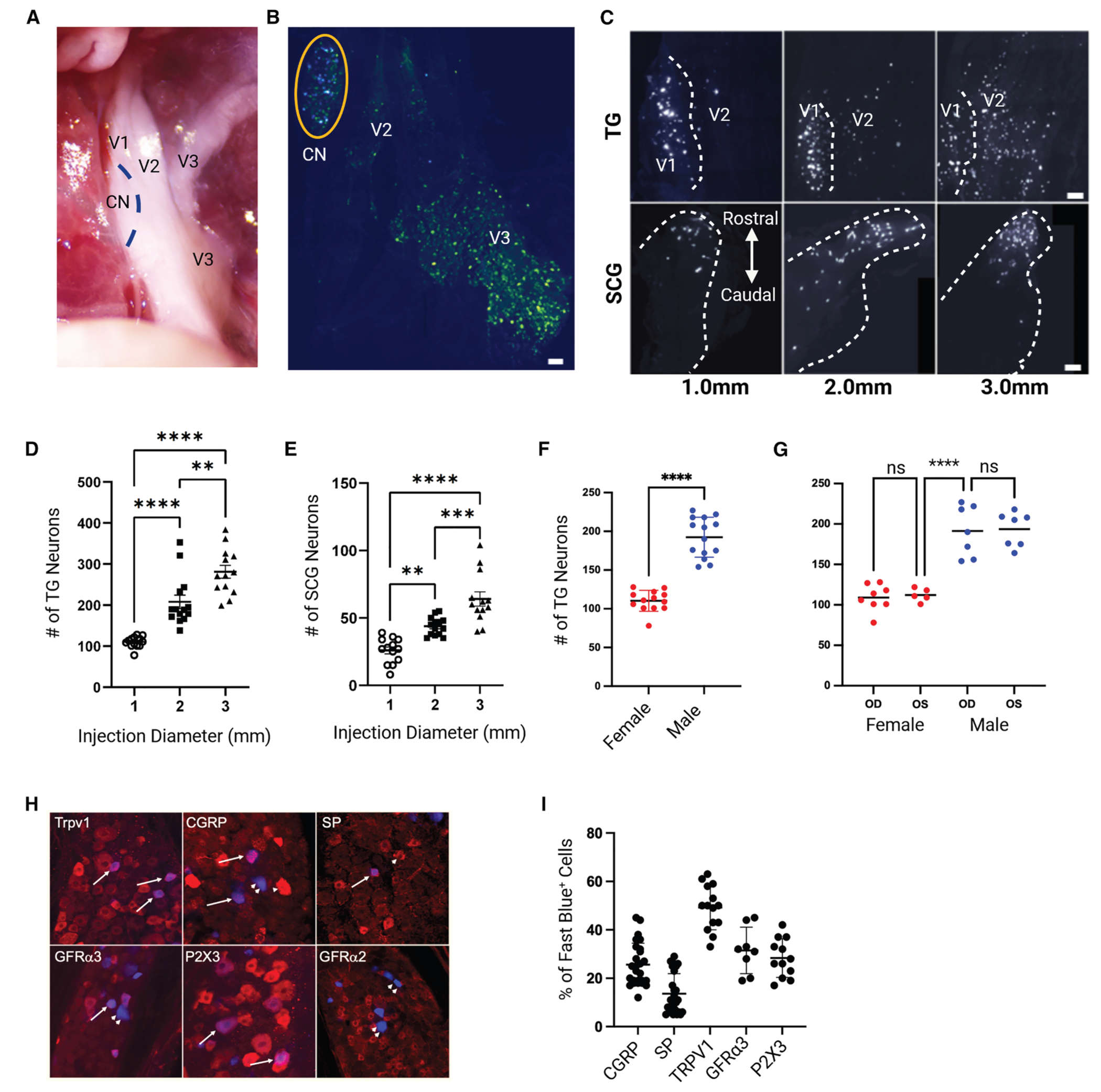
Characterization of the TG corneal niche (A) Dissection of ventral portion of the mouse skull showing the location of the three divisions of the right trigeminal ganglion (V1, V2, and V3) and the relation of the corneal niche (CN) to the three divisions. Unlike the human TG, there are two prominent branches that leave the skull from the V3. The CN is located in the most medial side of the V1. (B) Injection of fast blue (FB) into the cornea almost exclusively labels neurons in the CN. Anti-CGRP immunohistochemistry was used to show the location of peptidergic TG neurons. (C) Corneal injections larger than 1.0 mm label neurons outside of the V1 (top row). In the SCG (bottom row), the larger injections labeled more neurons, and these were seen in more caudal locations in the ganglion. (D) Quantification of the number of labeled neurons in TG following corneal injections with diameters from 1.0 to 3.0 mm. ****p < 0.0001 and **p = 0.001. (E) Quantification of the number of labeled neurons in SCG with different injection sizes. **p = 0.0021, ***p = 0.0005, and ****p < 0.0001. (F)There were almost twice as many back-labeled cells in the male mice compared to in the female mice (192 ± 6.9 vs. 110 ± 3.7; ****p < 0.0001). (G) There was no difference detected between the left and right eyes in either male or female mice (female mice OD [oculus dexter, right eye] = 109 ± 5.8 and OS = 112 ± 3.7; male mice, OD = 191 ± 12 and OS [oculus sinister, left eye] = 194 ± 8.0; ****p < 0.0001 and *p* > 0.5 both sexes). (H) Groups of mice were back labeled with FB, followed by immunohistochemical staining of whole ganglia with antibodies specific for peptidergic (CGRP, SP, Trpv1, and Gfrα3) and non-peptidergic (GFRα2 and P2X3) markers of afferent neurons. The arrowheads indicate the cells that were FB positive but did not stain for the indicated antibody. The arrows indicate back-labeled cells positively stained for each antibody. (I) Whole ganglia were imaged using confocal microscopy, and the number of labeled cells staining positive for each antibody was determined using Imaris software. 49% ± 2.5% of back-labeled cells were positive for Trpv1 staining, 26% ± 2.0% were positive for CGRP, 14.0% ± 2.0% were positive for SP, 32% ± 3.0% were positive for GFRα3, and 28.0% ± 2.0% were positive for P2X3. Less than 5.0% were positive for GFRα2. There was no sex difference in the percentage of neurons expressing any of the immunohistochemical markers (Table S1). For all graphs, one-way ANOVA with Tukey’s multiple comparison was the statistical test used for all comparisons. The bars represent the mean ± SEM.

The first step in identifying the mechanistic principles of sensory neuron homeostatic control was molecular phenotyping of corneal afferents. This analysis required back labeling followed by isolation of labeled afferents. We assumed that corneal afferents would be segregated within the V1 portion of the TG, as this gives rise to nerve branches that innervate the eye^20^ (Figure 1A). Injection of the retrograde dye fast blue (FB) into the cornea labeled cells in the most medial portion of the V1 (termed the corneal niche [CN]; Figures 1A and 1B). To determine the optimal parameters for selective labeling of corneal afferents, we employed different-sized injections (ranging from 1 to 3.0 mm in diameter) into the cornea. Photomontages of the injected eye showed that 1.0 mm injections limited the diffusion of dye to the cornea, whereas larger injections diffused into the surrounding tissues, including the conjunctiva (data not shown). Injections larger than 1.0 mm labeled cells in the V2 (maxillary) region of the TG, and the total number of back-labeled cells increased with increasing injection diameter (Figures 1C and 1D). Confirmation of dye spread outside of the cornea was confirmed by the appearance of increasing numbers of back-labeled neurons in the superior cervical ganglion (SCG), as sympathetic axons are normally restricted to the conjunctiva and do not enter the cornea^21^ (Figures 1C and 1E). Finally, using the 1.0-mm-diameter protocol, almost twice as many back-labeled cells were seen in the male mice compared to the female mice (with no difference between the right and left eyes), suggesting potential sex differences in corneal afferent sensitivity^22,23^ (Figures 1F and 1G). Furthermore, given the variability in neuronal counts being less than 15% in male and female mice, we were confident that our injection methodologies had sufficient precision for downstream studies. Combined, these results indicated that small, 1.0 mm injections were optimal for labeling corneal afferents, with minimal contamination by other afferent populations.

To separate corneal sensory neurons into broad classes (e.g., peptidergic or non-peptidergic), corneal peptidergic sensory neurons were identified based on the presence of immunostaining for CGRP, SP, Trpv1 (transient receptor potential cation channel type 1), and GFRα3 (GNDF [glial cell line-derived neurotrophic factor] family receptor alpha 3) (Figures 1H and 1I). Non-peptidergic neurons were identified based on expression of P2X3 and GFRα2 immunostaining. 58% of neurons expressed Trpv1, 21% of neurons were CGRP positive, and 18% were SP positive. Approximately 94% of CGRP-positive neurons were Trpv1 positive, whereas only 40% of SP-positive neurons expressed Trpv1 immunostaining. 55% of SP-positive neurons also expressed CGRP. The non-peptidergic marker P2X3 was expressed in 37% of corneal afferents, and 21% of these were also SP positive. Unlike cutaneous afferents, where 60%–70% express GFRα2, we found fewer than 5 back-labeled corneal afferents in any TG. Although these results were informative in that they indicated differences in the afferents between the cornea and other epithelial tissues like the skin, the identification of genes responsible for neuroimmune interactions required deeper transcriptomic analysis.

### Transcriptomic analysis of cornea-innervating TG nerves

**Figure 2.**
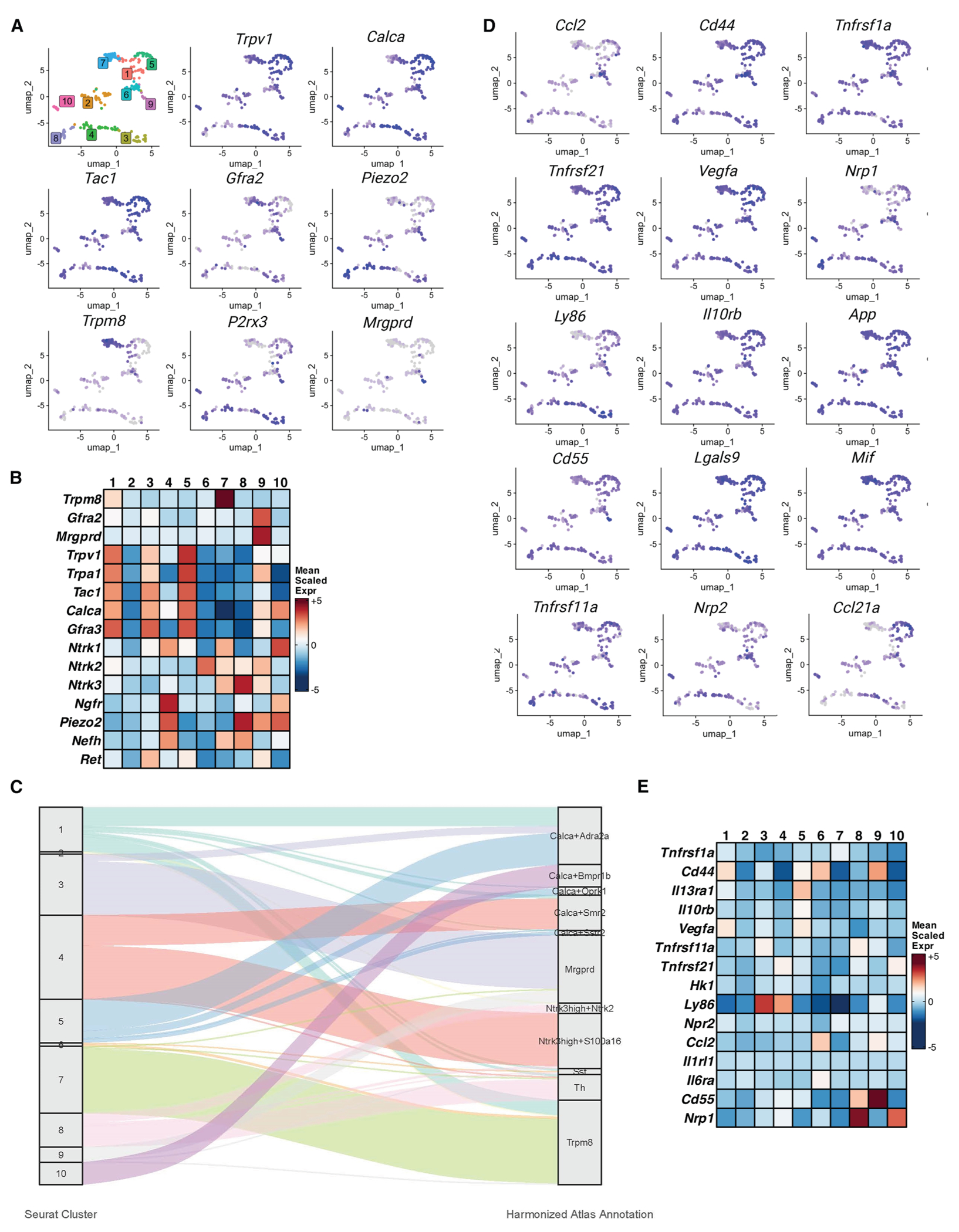
Transcriptomic analysis of TG sensory neurons (A) uMaps of transcripts used in previous studies to identify TG and DRG afferent clusters. Leiden cluster analysis was performed for scRNA-seq data from 254 handpicked, back-labeled corneal afferents from 6 male and 6 female mice. Cells had less than 10% mitochondrial transcripts and averaged 12,000 genes for this analysis. 10 different clusters were identified (1–10). (B) Heatmaps for neuron-specific transcripts that were expressed differentially in each cluster, i.e., genes with *p* < 0.05 significance for a given cluster. Transcripts associated with peptidergic neurons (e.g., *Trpv1*, *Calca*, *Tac1*, and *Gfra3*) were highly expressed in clusters 1, 3, 5, and 9. Clusters 4 and 8 were enriched for genes associated with myelinated low-threshold mechanoreceptors, including *Piezo2* and *Nefh*. Cluster 7 was distinguished by high levels of expression of the cool receptor *Trpm8*. (C) Sankey plot comparing the scRNA-seq datasets from the 254 handpicked neurons generated in this study to previously published datasets from full mouse TG (https://painseq.shinyapps.io/TG_Corneal_neurons/).^19,45^ (D) uMaps of corneal afferent transcripts that are normally associated with immune cells, including both ligands (e.g., *Ccl2*, *Vegfa*, *Ccl21a*, and *Mif*) and receptors (e.g., Tnfrsf-1a, −11a, and −21 and Il10rb). (E) Heatmap for immune-related transcripts that were differentially expressed in each cluster.

To assess the identity of cornea-innervating afferents, we hand-picked back-labeled neurons and performed scRNA-seq (Takara SMART-Seq Single-Cell Kit: 634473). Transcriptomics of handpicked, retrograde-labeled neurons, followed by Leiden analysis, identified 10 different afferent clusters (Figure 2; Tables S1 and S2). Importantly, we confirmed that our cells were not contaminated with glia, as counts for glial markers such as *Gfap*, *Ube2c*, *Cdc20*, *Birc5*, *Cdk1*, *Pbk*, and *Sox10* were virtually undetectable (Figure S1; Table S1). Similarly, the average number of genes per cell was 12,000, and less than 5% of RNA reads were mitochondrial, suggesting that our datasets were sufficient to define neuronal populations (Figure S1). As seen in previous studies of both TG and dorsal root ganglion (DFG) neurons, *Calca* was widely expressed, being found in all but 2 clusters (Figures 2A and 2B). *Trpv1*, the transcript that encodes the non-selective cation channel that is responsible for detecting noxious heat in a subset of cutaneous afferents,^24,25^ was expressed as broadly as *Calca*. The wide distribution of *Calca* overlaps extensively with *Tac1* (tachykinin precursor 1; the transcript that produces SP), *Trpa1* (a putative cold and mechanoreceptor^26–31^), and *GFRα3*. In cutaneous DRG neurons, all of these transcripts are typically seen in C-fibers, and based on this combination of genes and the one report that combined *ex vivo* physiology with single-cell transcriptomic analysis,^32^ they are likely to be peptidergic nociceptors, sensitive to noxious mechanical and/or heat stimuli. *Piezo2*, a low-threshold mechanosensitive channel,^33–35^ was most highly expressed in clusters 5 and 8–10. Cluster 8 also expressed high levels of *Ntrk3*, the receptor for Nft3 (neurotrophin-3) and *Nefh* (heavy-chain neurofilament), which, in cutaneous fibers, are restricted to A-fiber mechanoreceptors with a bias toward low-threshold afferents.^32,36–41^ Cluster 7 is one of the more discrete clusters and potentially functionally distinct in that virtually every cell expresses *Trpm8*, the transient receptor potential family member that detects temperatures between 18°C and 23°C.^12,42–44^ Transcriptomic analysis also showed that the levels of expression for *Trpv1*, *Calca* (transcript coding for CGRP), and *Tac1* (transcript coding for SP) were correlated, whereas markers for non-peptidergic neurons were not (Figure S1), suggesting the coordinated expression of these genes, and potentially their function, with respect to corneal homeostasis. Notably, cluster 9, which makes up fewer than 5% of sequenced neurons, expressed MrgprD—an itch-sensing neuron that terminates in the conjunctiva. The low frequency of these cells being detected by our scRNA-seq analyses suggested to us that our dataset included minimal leaching to the peripheral portions of the eye. Given that our analyses yielded 10 clusters of neurons, we next asked how these clusters compare to previously published datasets of sequenced neurons from the TG without consideration of the site of innervation. Therefore, using previously published resources,^19,45^ we anchored our populations of neurons with the hallmark genes of previously identified clusters of neurons from previously published TG atlases. Overall, despite differences in clustering patterns, it appears that the cornea does not exclude any specific types of neurons, as illustrated by the Sankey plots comparing the 10 clusters from this study to the 11 previously identified clusters (Figure 2C).

Each of the above genes has obvious functional potential to detect environmental signals (e.g., temperature changes, loss of moisture, and irritating particulates) that evoke protective reflexes (e.g., blinks and lachrymation). As noted, there is also a growing amount of literature showing potential crosstalk between sensory neurons and immune cells.^46^,^47^ Therefore, we examined our afferent transcriptomic database for the expression of chemokines, cytokines, and their receptors that were differentially expressed in individual clusters (Figures 2D and 2E). We found significant expression of immune-related transcripts, including *Vegfa* (vascular endothelial growth factor), *Ccl2* (also known as [aka] *Mcp1* [monocyte chemoattractant protein]), *Cd44* (a cell surface glycoprotein and adhesion molecule that interacts with macrophages and neutrophils and plays role in T cell homing), *Cd55* (as a secreted protein important in the complement cascade), *Ly86* (cooperates with Toll-like receptor [TLR]4 [also expressed in a subset of corneal afferents] to regulate innate immune responses), and *Nrp1* (neuropilin; identified as playing a role in neural development and as inhibitory checkpoint proteins). Other transcripts that encode genes that are important for responding to immune cytokines were widely expressed and found in all clusters. These included *Tnfrsf1a* (tumor necrosis factor [TNF] receptor 1), receptors for interferon (IFN) alpha and gamma (*Ifnar 1&2* and *Ifngr 1&2*), and the interleukin (IL)-6 signal transducer (*IL6st*, aka *CD130*) that is downstream of binding cytokines, including IL-11, IL-6, CNTF (ciliary neurotrophic factor), and LIF (leukemia inhibitory factor).

### Predictions of neuro-immune interactions at the ocular surface and within the TG

**Figure 3.**
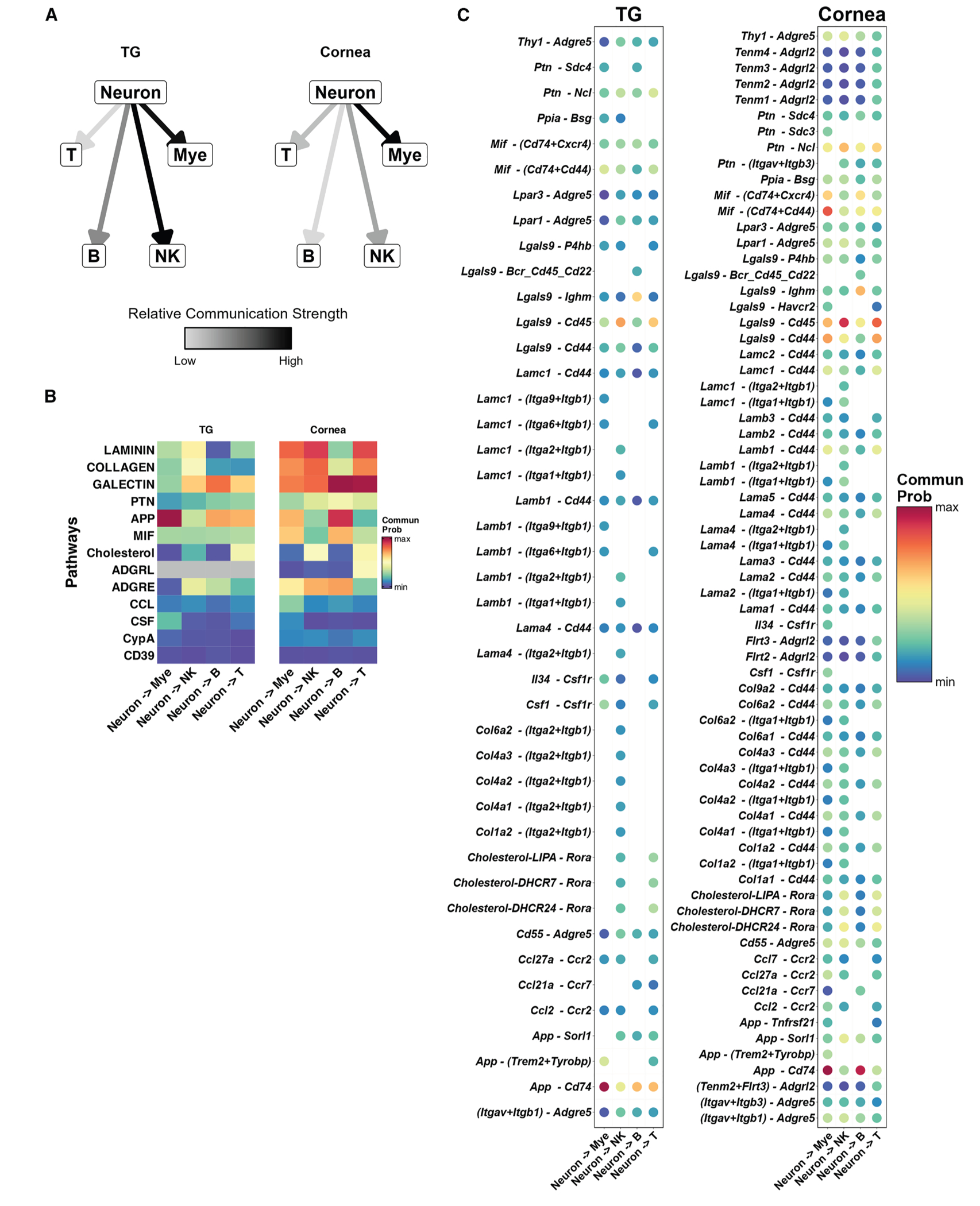
Potential neuron-to-immune cell interactions in the mouse trigeminal ganglion and cornea (A) Relative communication strength for signaling from mouse corneal afferents to immune cells in the TG and cornea. (B) Relative communication probabilities (per anatomic location) for potential neuron-to-immune cell signaling pathways (gray color indicates no significant interaction detected). (C) Relative communication probabilities of significant neuronal ligand and immune cell receptor pairs (*p* < 0.05 by permutation testing). Most neuronally expressed genes that showed significant interactions with immune cells had more than one pairing with immune cell-expressed molecules, and these pathways were expressed in more than one immune cell type.

Because recent data support the active communication between sensory neurons and immune cells located at the sensory ganglia and periphery,^1,48,49^ we sought to perform scRNA-seq on immune cells from the cornea and the TG (Figure S2). For scRNA-seq, due to the nature of quiescent RNA in neutrophils, we used fluorescence-activated cell sorting (FACS) to isolate CD45^+^Ly6G^−^ immune cells from the TG and cornea. Examination of the cornea showed that of the immune cells present, myeloid cells were dominant (45%), followed by T (33%), natural killer (NK) (13%), and B (9%) cells (Figure S2). Additionally, within the non-neutrophil immune compartment ofthe TG, scRNA-seq analysis identified a large myeloid cell population (63.6% of all CD45^+^ cells), with B, NK, and T cells comprising the remainder (29.7%, 3.5%, and 3.2%, respectively) (Figure S2). With these data, we used the CellChat analysis tool^50–52^ to identify potential intercellular interactions in an unbiased manner. We first explored potential cell crosstalk where neurons were the ‘‘output’’ and immune cells were the ‘‘input’’ (Figure 3). CellChat analysis of corneal afferent neurons and TG immune cells found intercellular communication to be enriched most strongly for neuron-to-myeloid and neuron-to-NK cells (Figure 3A). Additionally, CellChat analysis using transcriptomic data from corneal afferents and corneal immune cells found that the strongest intercellular signal pairing was for neurons and myeloid cells (Figure 3A). Conversely, analysis in which the immune cells were the output found that the strongest interactions with neurons were also between myeloid and NK cells (Figure S3). In the TG, interrogation at the level of signaling pathways found the strongest neuron-to-immune communication probabilities for neuronally expressed *lgals9* (galectin), *Mif* (macrophage migration inhibitory factor), *Ccl2*, *App* (amyloid precursor protein), *Adgre* (adhesion GPCR E1), and *Ptn* (pleiotropin) (Figures 3B and 3C). Many of the same neuronal genes that were recognized as having potentially significant interactions with TG immune cells were identified for corneal immune cells (Figures 3B and 3C). These included *lgals9*, *Mif*, *App*, *Adgre*, *Ccl2*, and *Ptn*. Additional neuronally expressed genes with strong immune cell pairings for corneal immune cells included *Lamb3* (laminin subunit beta 3), *Lamc1* (laminin subunit gamma 1), *Nrxn1* (neurexin 1), *Ppia* (peptidylprolyl peptidase 1), and *Thbs1* (thrombospondin).

### Corneal afferents mediate early anti-viral immunity

**Figure 4.**
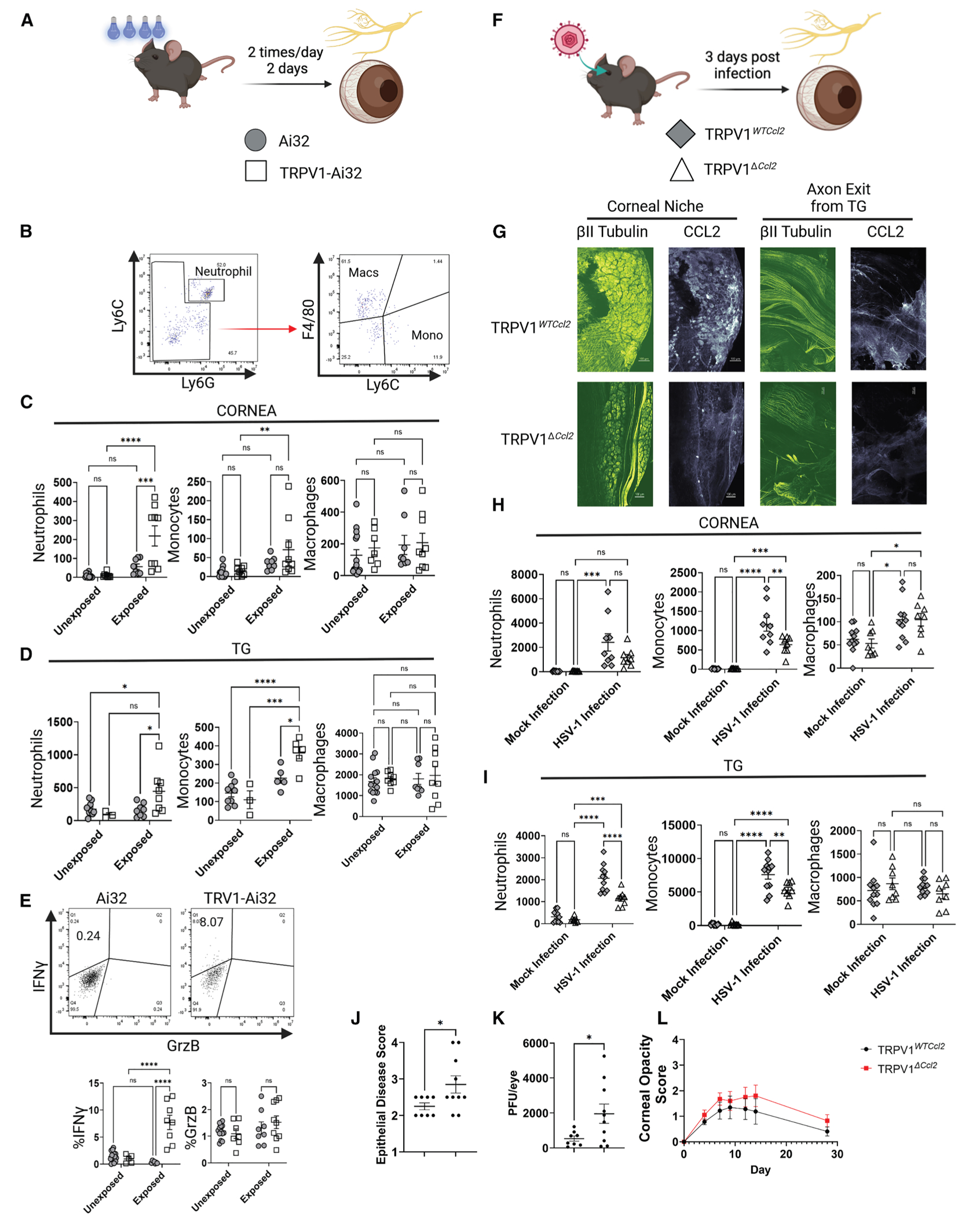
Corneal afferents modulate tissue inflammatory responses (A) Ai32 or TRPV1-Ai32 mice were anesthetized with isofluorane for 1 h, two times a day, and exposed to blue (473 nm) LED light (ca. 2 mW). On day 3, the corneas and TG were harvested from mice, dispersed into single-cell suspensions, and analyzed by flow cytometry. (B) Representative gating strategy for neutrophils, macrophages (Macs), and monocytes (Monos). (C) Quantification of neutrophils, Monos, and Macs isolated from cornea. **p = 0.0065, ***p = 0.0002, and ****p < 0.0001. (D) Quantification of neutrophils, Monos, and Macs isolated from TG.*p = 0.0184–0.0359, ***p = 0.0003, and ****p < 0.0001. (E) NK cells in TG were analyzed for *in situ* production of IFNγ and granzyme B (GrzB). ****p < 0.0001. (F) The eyes of TRPV1^*WT CCL2*^ or TRPV1^*ΔCCL2*^ mice were scarified, and one eye was infected with 1 × 10^5^ plaque-forming units (PFUs) of HSV-1 (KOS strain). PBS was applied to the contralateral eye. (G) On day 3 post-infection, mice were sacrificed, and TGs were analyzed for CCL2 expression using a fluorescent antibody against the fluorescent protein, RFP, and immunohistochemistry (IHC). Full TGs were imaged, and the images represent the corneal niche and the area where axons exit the TG and traverse to the eye. (H and I) In parallel, myeloid cells from the (H) cornea (*p = 0.0237–0.0499, **p = 0.0037, ***p = 0.0003–0.0008, and ****p < 0.0001) or (I) TG (**p = 0.0040, ***p = 0.0001–0.0004, and ****p < 0.0001) were quantified and analyzed by flow cytometry, as seen in the representative flow plots in (B). T, B, and NK cell data are provided in Figure S4. The clinical scores and viral clearance from the cornea were measured longitudinally in infected mice. (J) Epithelial disease was assessed 2 days post-infection. (*p = 0.0367). (K) Viral PFU on day 4 post-infection was measured using corneal swabs (*p = 0.0316). (L) Corneal opacity scores were measured from initial infection to 28 days post-infection. All data points present individual mice from three independent experiments for (C)–(E) and two independent experiments for (G)–(L). Statistical significance for (C)–(E), (H), and (I) was determined using a two-way ANOVA, and statistical significance for (J) and (K) was determined using an unpaired *t* test with Welch’s comparison. The bars represent the mean ± SEM.

Knowing that neuronal activity is increased in the presence of infectious or traumatic insult and that corneal insults produced changes in immune cells, we asked whether increased neuronal activity alone was sufficient to alter the number and/or phenotype of these cells in the TG and cornea. This was accomplished by expressing channelrhodopsin 2 (ChR2) via the *Trpv1* promoter in corneal afferents (aka *Trpv1-Ai32* mice), followed by stimulation with 473 nm light-emitting diode (LED) lights (2 mW) for 1 h twice a day for 2 days. We have previously used this approach to successfully activate primary afferents innervating the glabrous skin of footpads in awake mice in a conditioned place assay^53^ (Figure 4A). Following exposure to blue light, the *Trpv1-Ai32* mice exhibited an increase in neutrophils (gating strategy in Figure 4B) in the TG and cornea (Figures 4C and 4D). In addition, monocytes were increased in both the cornea and TG in *Trpv1-Ai32* mice with light exposure (Figures 4C and 4D). No changes were seen in the number of T, B, or NK cells (data not shown). The only caveat to note is that the numbers of both neutrophils and monocytes were also increased, albeit to a lesser extent, in *Trpv1-Ai32* mice in the absence of LED exposure compared to wild-type mice. This may reflect the ability of 473 nm light present in normal fluorescent light (that can vary from 50 to 110 μW in our vivarium) to activate Ai32 in corneal afferents. Finally, TG NK cells exhibited an increase in IFNγ but only in *Trpv1-Ai32* mice exposed to blue light (Figure 4E). Interestingly, there was no change detected in the number of macrophages in the TG and cornea regardless of genotype or treatment (Figures 4C and 4D), suggesting that the brief exposure to the LED light initiated an immune response typical of the earliest stages of inflammation.

To assess whether CellChat targets were physiologically relevant for the ocular surface, we interrogated the role of neuronal-expressed *Ccl2* as a potential modulator of myeloid, NK, and T cells. Specifically, we wanted to determine if neuronal-expressed Ccl2 could be responsible for the recruitment of immune cells after a viral infection, a common clinical problem that can lead to corneal scarring. Given the importance of Ccl2 during primary stages of HSV-1 infection,^54–56^ we sought to examine whether corneal sensory afferents were responsible for the initial recruitment of immune cells. Trpv1-cre mice (the same as those used to generate the *Trpv1-Ai32* mice) were crossed with Ccl2floxed mice, and the resulting knockout mice (*Trpv1*^*ΔCcl2*^) and littermate controls were ocularly infected with HSV in one eye, with the contralateral eye serving as the control (Figure 4F). At 3 days post-infection, *Trpv1*^*ΔCcl2*^ mice had marked reductions in the expression of *Ccl2* in the CN and the axons exiting the TG that would eventually innervate the cornea (Figure 4G). Notably, Ccl2 was lost in all TRPV1-expressing neurons in the TG, not just those neurons within the CN. Therefore, these data illustrate that Trpv1-expressing neurons produce CCL2 after HSV-1 infection. Additionally, both genotypes exhibited increases in neutrophils and monocytes in the cornea and TG (Figures 4H and 4I). Despite no differences existing between the control TGs of both genotypes, fewer monocytes were recruited to the cornea in the *Trpv1*^*ΔCcl2*^ compared to the control mice. In the cornea, neutrophil trended lower in *Trpv1*^*ΔCcl2*^ mice, but this difference was not significant (Figure 4H). There were also notable reductions in neutrophil and monocyte migration into the TG of *Trpv1*^*ΔCcl2*^ mice (Figure 4I). The effect of the diminished inflammatory response in *Trpv1*^*ΔCcl2*^ mice was observed in a more severe epithelial disease score 2 days post-infection, and a significantly higher viral burden was seen 4 days post-infection (Figures 4J and 4K). While there was a trend toward a more severe corneal opacity through latency, statistically significant differences in disease were not observed (Figure 4L). Given the kinetics of chemokine expression and the many redundancies in place within the immune system, it is unsurprising that we did not observe lasting effects of neuronal CCL2 deficiency. Overall, we attribute the differences during acute infection to the impaired recruitment of myeloid-derived cells within 3 days post-infection because we did not observe any other differences in immune cell compartments during acute infection (Figure S4). Together, our results indicate that the release of CCL2 from sensory neurons after neurotrophic viral infection is an early mediator of anti-viral immunity and contributes to the first wave of myeloid cell recruitment, which contributes to the initial clearance of the virus from the cornea.

## DISCUSSION

These experiments employed transcriptomics that revealed that corneal afferents, unlike cutaneous afferents, are dominated by peptidergic afferents, similar to visceral structures, such as the colon and bladder,^6,8^ and like other sensory neurons, they express a wide range of genes capable of interacting with immune cells. Deletion of a specific neuronally expressed chemokine (*Ccl2*) identified by the unbiased CellChat analysis resulted in the predicted changes with respect to types of immune cells altered in both the TG and cornea in response to inflammation. Similarly, those immune cells predicted to have the greatest strength of interaction with corneal afferents (myeloid and NK cells) responded to optogenetic activation of corneal afferents. Based on these observations, it is easy to propose a scenario in which corneal afferents choreograph an immune response that starts in the cornea and then travels to the TG and ultimately to the central nervous system to be potentiated by protective reflexes, including autonomic changes that protect the eye. However, this is likely an oversimplification of the complex interactions that protect barrier tissues such as the cornea. More serious injuries of the cornea initiate a series of responses that include a robust response by adaptive immune cells, withdrawal of sensory fibers, and growth of sympathetic fibers that can be accompanied by both lymphatic and blood vessels. In this context, it should be noted that our transcriptomic profiling indicated that sensory neurons express high levels of *Vegf*, a cytokine critical for the growth of blood vessels. Moreover, complete healing is usually accompanied by retraction of these anatomical elements and restoration of sensory innervation.^57^ The multicellular interactions supporting corneal homeostasis should be no surprise given that these cell types evolved simultaneously, and as they are present during the evolution of complicated organs such as the eye, coordinated responses to injury would provide a clear selective advantage. Thus, it might be useful to stop thinking about ‘‘neuroimmune’’ interactions as auxiliary roles for peripheral neurons and immune cells; the results presented here indicate that both sensory neurons and immune cells sense and respond to changes in their environment and that, through their crosstalk, they are inextricably linked as mediators of tissue homeostasis.

### Limitations of the study

Our study establishes a communication network between corneal nerves and immune cells, both at the neuronal termini and cell bodies. This required a systematic approach that entailed labeling, isolating, and sequencing neurons, FACS isolating cornea and TG immune cells, and computational analytics. Limitations to our analyses exist at the level of labeling, where less than 5% of sequenced neurons appeared to be classified as terminating at the conjunctival region, given the expression of ‘‘itch’’ channels, MrgprA3 and MrgprD. The reason for these apparent contaminants could be the leaching of the cell tracing dye from the cornea to the limbal region. On the level of immune cell sequencing, we excluded neutrophils during our FACS isolation step, given their transcriptional quiescence, relatively low numbers during steady state, and their potential to be associated with blood contamination. Finally, the Trpv1^*ΔCcl2*^ mouse used in the final study lacked *Ccl2* in all TRPV1-expressing neurons. This detail allows for the possibility that the observed effects on HSV-1 infection may not be restricted to CCL2 produced in the CN of the TG but may rely on *Ccl2* expression in other areas of the TG or, more broadly, systemic release of *Ccl2* from other *Trpv1*-expressing neurons throughout the body.

## RESOURCE AVAILABILITY

### Lead contact

Requests for further information and resources should be directed to and will be fulfilled by the lead contact, Anthony St. Leger (anthony.stleger@pitt.edu).

### Materials availability

This study did not generate new unique reagents.

### Data and code availability

- Neuronal datasets have been deposited at https://painseq.shinyapps.io/TG_Corneal_neurons/ and are publicly available as of the date of publication.
- All raw data, including neuronal and immune cell scRNA-seq datasets, have been deposited at https://doi.org/10.5281/zenodo.17364394. The accession code is pending at the time of publication. It will be supplied once it is available.
- All data reported in this paper will be shared by the lead contact upon request.
- Any additional information required to reanalyze the data reported in this paper is available from the lead contact upon request.

## STAR★METHODS

### EXPERIMENTAL MODEL AND STUDY PARTICIPANT DETAILS

Please list here under separate headings all the experimental models/study participants (animals, human participants, plants, microbe strains, cell lines, primary cell cultures) used in the study. For each model, provide information related to their species/strain, genotype, age/developmental stage, sex (and gender, ancestry, race, and ethnicity if reported for human studies), maintenance, and care, including institutional permission and oversight information for the experimental animal/human participant study. The influence (or association) of sex, gender, or both on the results of the study must be reported. In cases where it cannot, authors should discuss this as a limitation to their research’s generalizability.

### METHOD DETAILS

#### Animals

Male and female wild-type C57BL/6 mice purchased from Jackson Laboratories (Bar Harbor, ME, USA) were housed in the Animal Resource Facility at the University of Pittsburgh Medical Center (Pittsburgh, PA, USA) and used at 6–8 weeks of age in all experiments. To produce mice expressing channelrhodopsin (ChR2) under the Trpv1 promoter floxed ChR2 mice (Jax #012569) were crossed with mice expressing Cre recombinase under the TRPV1 promoter (Jax #017769). To generate conditional TRPV1^*ΔCcl2*^ mice, B6.129-Trpv1^tm1(cre)Bbm/J^ (JAX Strain: 017769) were crossed with B6.Cg-Ccl2^tm1.1Pame/J^ (JAX Strain: 016849). The research protocols and experimental procedures were reviewed and approved by the University of Pittsburgh Institutional Animal Care and Use Committee (Protocol number: 25117637 (Formerly 22112133)), ensuring full compliance with the ARVO Statement for the Use of Animals in Ophthalmic and Vision Research.

#### Intracorneal stromal injection

Mice were anesthetized by intraperitoneal injection of ketamine hydrochloride (100 mg/kg body weight) and xylazine (0.1 mg/kg body weight) in Hanks’ balanced salt solution. A 31-gauge needle was used to create a short tunnel beneath the basement membrane in the central cornea. The needle of a microinjection syringe was then inserted into this tunnel to inject the 0.01% fluorescent tracing dye into the corneal stroma. Transient opaqueness of the cornea occurred immediately following the injection. Consequently, the area of corneal injection was confined to be either a 1 × 1mm^2^, 2 × 2mm^2^, or 3 × 3mm^2^ region.

#### HSV-1 infection

Mice were anesthetized by intraperitoneal injection of ketamine hydrochloride (100mg/kg body weight) and xylazine (0.1 mg/kg body weight) in HBSS. After mice were confirmed to be unresponsive (toe pinch), the cornea was scratched in a hatched pattern 12 times with a 31-gauge needle. Then, 3μL of either RPMI alone (mock infection) or 1 × 10^5^ PFU of HSV-1 KOS strain (HSV-1 infection) in RPMI was applied to the cornea. After infection, mice were then given atimapezole (6.25 × 10^−3^ mg/mouse) to reverse the effects of ketamine/xylazine mixture. Three days after infection, mice were sacrificed, and tissues were processed accordingly.

At 2 days post infection, epithelial disease was evaluated by fluorescein staining and graded in a masked manner as follows: 0, normal cornea; 1, punctate epithelial lesions; 2, small dendritic lesions; 2.5, large dendritic lesions; 3, small geographic lesions involving <25% of the corneal surface; 4, large geographic lesions involving >25% of the corneal suface.

For viral burden, mouse corneas were swabbed with sterile Weck-Cel surgical spears (Beaver Visitec, Waltham, MA) at 4 days post infection, and spears were placed in 0.5 ml RMPI and frozen at −80°C until assayed. Dilutions of samples were added to confluent Vero cells, incubated for 1 h at 37°C, and overlaid with 0.5% methylcellulose. The cultures were incubated for 48 h, fixed with formalin, and stained with crystal violet, and viral plaques were counted with the aid of a dissecting microscope.

#### Immunohistochemistry

For IHC analysis, tissue was process as described in Yun et al., 2021.^58^ Briefly, following dissection, tissues were fixed at room temperature for 1 h in a solution of 1.3% paraformaldehyde in PBS. Subsequently, tissues underwent five washes in PBS and were permeabilized in a 1% Triton X-100 solution in PBS for 60 min at room temperature. Tissues were then blocked using either 20% goat serum or donkey serum (Cedarlane, Burlington, NC, USA) in a blocking buffer (consisting of 0.3% Triton X-100 and 0.1% Tween 20 in PBS) for 1 h. Next, the tissues were exposed to a cocktail of primary antibodies (100μL) at room temperature for 2 h, with an additional overnight incubation at 4°C. The corneas then underwent five 5-min washes with a wash buffer (0.1% Tween 20 in PBS). Subsequently, the corneas were incubated in a cocktail of secondary antibodies (100μL) in blocking buffer at room temperature for 2 h. After undergoing five 10-min washes with the wash buffer, the tissues were mounted on slides and allowed to dry at 4°C for at least 12 h before imaging.

#### Confocal microscopy

Stitched Z-stacks covering the entire tissue whole mounts were obtained using an OLYMPUS BX61 motorized upright Fluoview 1200 laser scanning confocal microscope equipped with either an ×20 or a 60x oil (numerical aperture, 0.85) objective lens and an automated stage. Each z stack were composed of images separated by 3μm to ensure that all labeled sensory neurons were captured. The z stack images were saved in the native Olympus Image Binary (OIB) format and subsequently stitched together using FV10-ASW 2.0 software (Olympus Life Science, Tokyo, Japan). Imaris (version 10.0.1, Oxford Instruments, https://imaris.oxinst.com) software was used to analyze the confocal images. To quantify the neurons, a plug-in of ‘‘Labkit for Pixel Classification’’ was applied in the ‘‘Surfaces’’ when quantifying the neurons.

#### Collection of corneal afferents

Corneas were injected with fluorescently-tagged WGA (Alexa Fluor 555 or 488) in the center of the cornea to cover ca. 1.0 mm^2^. In all cases, both eyes were injected and ganglia from both sides were combined and treated as a single sample. Equal numbers of male and female mice were used in these experiments. Three days post-injection, mice were deeply anesthetized, perfused with HBSS and trigeminal ganglia removed. Trigeminal neuron dissociation was accomplished as described previously.^59^ Briefly, following dissection, ganglia were treated with a combination of cysteine, papain, collagenase type II, and dispase type II, followed by trituration, centrifugation through a percoll gradient and plated in DMEM on poly-*d*-lysine/laminin-coated coverslips in 35 mm × 10 mm petri dishes. Two hours later, cells were visualized on an inverted microscope with a heated stage and hand-picked using pulled glass microelectrodes (similar to patch clamp electrodes) fitted into a patch clamp electrode holder attached to 3-axis micromanipulator. Individual cells were sucked into the pipette using negative pressure, transferred to tubes containing 12μL of lysis buffer (Smart Seq V4, Takara Bio) and frozen at −80C. 24 cells were picked up from each mouse resulting in 288 samples that were submitted for mRNA library production and analysis.

#### Full-length single-cell RNA-seq library preparation and sequencing of corneal afferents

Full-length cDNA was generated from single cells with the Takara SMART-Seq Single-cell kit (Takara: 634473) according to the manufacturer’s instructions. Briefly, tubes with cells were defrosted at 72°C for 3 min then held on ice prior to cDNA generation using 17 cycles for SMART whole genome amplification. cDNA was assessed for quality using an Agilent Fragment Analyzer 5300 prior to libraries being generated by tagmentation with the SMART-Seq Library Prep Kit (Takara: R400747) using Takara Unique Dual Index Kits (Takara: 634753–634755) and 15 cycles of amplification. Libraries from each individually collected cell were pooled by animal (in equal volume) prior to library cleanup using Agencourt Ampure XP beads. Library pool quantification and assessment was done using a Qubit FLEX fluorometer and an Agilent Fragment Analyzer 5300. Libraries were normalized and pooled to 2nM by calculating the concentration based on the fragment size (base pairs) and the concentration (ng/μL) of the libraries. Sequencing was performed on an Illumina NextSeq 2000, using a P2 300 flow cell. The pooled library was loaded at 750 pM. Sequencing was carried out 2 × 151 bp, with a target of 1 million reads per sample. Sequencing data was demultiplexed by the on-board Illumina DRAGEN FASTQ Generation software (v3.10.12). Of the 284 samples, 254 produced high quality RNAseq libraries (ca. 88% of all samples submitted). Transcript abundance was performed with Kallisto (v0.46.1) using an index based on the Ensembl v96 reference transcriptome for mus musculus.^60^

#### Single-cell preparation of corneal and TG immune cells

Trigeminal ganglia and corneas were harvested from treatment naive C57bl/6 mice (6 replicates in total across two dates for the trigeminal ganglia and 3 replications from one date for the corneas). Samples were processed for single cell suspension by first mincing (mechanical digestion) then incubating at 37°C for 1 h in an RPMI +10% FBS + collagenase (Type I, 420U/mL final concentration). After incubation, cells were dispersed into single cell suspensions by triturating with a P200 pipet. Then, cells were stained with antibodies against CD45, Ly6G, CD326 (EpCam). Then, CD45^+^Ly6G^−^ EpCam^-^ cells were isolated via FACS-sorting using a Sony MA900 cell sorter. Single-cell capture was performed on sorted cells using the 10x Genomics platform (3′ assay). Libraries were sequenced using the Illumina platform. Alignment, filtering, barcode counting, and unique molecular identifier counting were performed using the 10x Genomics CellRanger pipeline.

#### Transcriptomic analysis of neuroimmune interactome

Neuronal and immune single-cell analysis was performed in R using Seurat, with normalization and variance stabilization of count data using scTransform v2^61,62^. For neuronal data, all hand-picked labeled cells were included in analyses. Corneal and TG immune cells were filtered to exclude cells with fewer than 200 gene features, more than 10% mitochondrial DNA, and a feature or total gene counts above the 95^th^ percentile (for doublet exclusion). Dimensional reduction was performed using principal component analysis (dimensions = 50) followed by the UMAP (Uniform Manifold Approximation and Projection) algorithm. Unsupervised clustering was performed using Seurat’s shared nearest neighbor (FindNeighbors) algorithm followed by Leiden clustering at a resolution of 1.0. Immune cell clusters were annotated to immune cell type based on expression of the following marker genes: Myeloid - *Adgre1*, *Cd83*, *Cd68*; B - *Pax5*, *Cd19*, *Ighm*; NK – *Klrb1c*, *Klrk1*; T - *Cd3d*. Ligand-receptor analysis between trigeminal ganglia or cornea CD45^+^ immune cells and corneal afferents was performed using the CellChat v2 package with methodologies as previously described for determination of ligand-receptor interaction communication probabilities.^52^ Relative cell-cell signaling pathway and overall cell-cell communication probabilities were calculated by summarizing the communication probabilities of all ligands-receptors interactions associated with each signaling pathway or cell-cell interaction as previously described. Ligand-receptor pathways with a communication probability in the top 10% were selected for reporting and downstream analysis, and individual ligand-receptor pairs from these pathways with a scaled relative communication probability *Z* score of 0.5 or higher were reported.

#### Flow cytometric analysis of immune cells in the trigeminal ganglion

TGs or corneas were excised from mice cardiac perfused with 0.9% saline after being heavily anesthetized with an i.p. injection of ketamine and xylazine (1g/kg of ketamine, and 1 mg/kg of xylazine). Full TGs or minced corneas were chemically digested in RPMI (Bio Whittaker) containing 10% FBS (Atlanta Biologicals) and 400 U/ml collagenase type I (Sigma-Aldrich) for 1 h at 37°C. TGs or corneas were dispersed into single-cell suspensions by trituration through a p-200 pipette tip. Samples were then spun down through a blue-filter capped FACS tube (Falcon). 0.5 TGs or 0.5 corneas were stained with Ghost 780 viability dye (Tonbo) followed by being stained with fluorescent antibodies against the proteins listed below. All surface stain antibodies were diluted in brilliant stain buffer (BioLegend). Samples were fixed and permeabilized using Cytofix/Cytoperm Fixation/Permeabilization kit (BD Biosciences). All samples were assessed using a Cytoflex LX (Beckman Coulter), and data were analyzed using FlowJo v10.1 software (BD Biosciences).

**Table:** 

Monocyte panel	T cell/NK cell	T cell/B cell
CD45.2 BV785	CD45.2 BV785	CD45.2 BV785
Ly6C FITC	TCR alpha PE	CD8α FITC
CD11b PE Dazzle 594	NKG2D PE Dazzle 594	CD4 PE
CD11c BV421	NK1.1 PECy7	γδ TCR PECy7
I-A/I-E BV510		TCRβ BV421
CCR2 BV605	IL-10 APC (*Intracellular*)	CD19 BV510
Ly6G BV650	IFN gamma FITC (*Intracellular*)	B220 APC
F4/80 PE-Cy7	Granzyme B BV421 (*Intracellular*)	
IL-10 APC (*Intracellular*)		
VEGF PE (*Intracellular*)		

#### Optogenetic activation of corneal afferents

For optogenetic activation of corneal afferents, mice constitutively expressing channelrhodopsin (ChR2; Jax cat. # 012569) after being bred to mice expressing cre-recombinase under the Trpv1 promoter (Jax cat # 017769) were anesthetized in an induction chamber and then moved to a manifold with 6 nose cones and administered 2% isoflurane (vaporized with 95% O_2_/5% CO_2_). Once a steady level of anesthesia was reached, a panel of LED emitting blue light we placed over the mice. Within the nosed cone the 2mW of blue light was measured using a hand-held light meter. Mice were exposed to the blue light for 1hr and returned to their home cage for 2 h, followed by repeated treatment. Mice were treated for two consecutive days and then processed for immune cell analysis in the cornea and the TG on day 3.

### QUANTIFICATION AND STATISTICAL ANALYSIS

All values are presented as mean ± SEM. The normality of each set of data were checked by D’Agostino & Pearson normality test with GraphPad Prism 10. (https://www.graphpad.com/quickcalcs/grubbs1/). All experiments using transgenic mice expressing ChR2 or in which we deleted the *Ccl2* gene contained four groups and were statistically compared using two-Way ANOVA with a Tukey’s Multiple Comparison test. Differences were considered to be statistically significant at *p* < 0.05. For experiments determining the number of back-labeled cells in the TG and SCG a Mann Whitney test was used to determine significance (*p* < 0.5).

## Supplementary Material

1

2

3

SUPPLEMENTAL INFORMATION

Supplemental information can be found online at https://doi.org/10.1016/j.celrep.2025.116693.

## Figures and Tables

**KEY RESOURCES TABLE:** 

REAGENT or RESOURCE	SOURCE	IDENTIFIER
Antibodies		
B220 APC	eBioscience	RRID:AB_469395
CCR2 BV605	BioLegend	RRID:AB_2721549
CD11b PEDazzle 594	BioLegend	RRID:AB_2563648
CD11c BV421	BioLegend	RRID:AB_2563099
CD4 PE	BD Pharmingen	RRID:AB_394585
CD8a AF488	BD Pharmingen	RRID:AB_396780
CD45.2 BV785	BioLegend	RRID:AB_2562604
F4/80 PE-Cy7	BioLegend	RRID:AB_893478
γδTCR PE-Cy7	BioLegend	RRID:AB_11203530
Granzyme B BV421	BD Horizon	RRID:AB_2738175
I-A/I-E BV510	BioLegend	RRID:AB_2734168
IFNγ FITC	BD Pharmingen	RRID:AB_395375
IL-10 APC	BD Pharmingen	RRID:AB_398558
Ly6C FITC	BD Pharmingen	RRID:AB_394628
Ly6G BV650	BioLegend	RRID:AB_2565881
NK1.1 PE-Cy7	eBioscience	RRID:AB_469665
TCRβ BV421	BD Horizon	RRID:AB_2737830
VEGF PE	Santa Cruz	RRID:AB_628430
NKG2D PE Dazzle 594	BioLegend	RRID:AB_2728148
Rat Anti-Substance P	BD Pharmingen	RRID:AB_396357
Anti-βIII Tubulin Rabbit Polyclonal Antibody	Abcam	RRID:AB_444319
Anti-Tyrosine Rabbit Polyclonal Antibody	Abcam	RRID:AB_1524535
Anti-mGFRα3 Affinity Purified Goat IgG	R & D Systems	RRID:AB_2110295
P2X3 Polyclonal Antibody (Rabbit/IgG)	Thermo Fisher Scientific (Invitrogen)	RRID:AB_2900342
Anti-TRPV1 (VR1) Polyclonal antibody (Rabbit)	Alomone Labs	RRID:AB_2313819
Anti-CGRP Goat polyclonal antibody	Abcam	RRID:AB_725807
Anti-h/mGFRα−2 Affinity Purified Goat IgG	R&D Systems	RRID: AB_2294621
Mouse anti-RFP Monoclonal Antibody	Chromotek	RRID: AB_2631395
Donkey anti-goat IgG (H + L) Alexa Fluor 633	Molecular Probes	RRID:AB_10562400
Donkey anti-rabbit IgG (H + L) Alexa Fluor 488	Thermo Fisher Scientific (Invitrogen)	RRID:AB_2534102
Donkey anti-rabbit IgG (H + L) Alexa Fluor 647	Thermo Fisher Scientific (Invitrogen)	RRID:AB_2536183
Donkey anti-goat IgG (H + L) Alexa Fluor 546	Life Technologies	RRID:AB_2534103
Donkey anti-rat IgG (H + L) Alexa Fluor 647	Invitrogen	RRID:AB_2893138
Donkey anti-rat IgG (H + L) Alexa Fluor 488	Thermo Fisher Scientific (Invitrogen)	RRID:AB_2535794
Donkey anti-rat IgG (H + L) Alexa Fluor 568	Abcam	RRID:AB_2636887
Bacterial and virus strains		
Herpes Simplex Virus type 1 (KOS Strain)	Paul K. Kinchington	N/A
Critical commercial assays		
SMART-Seq (R) mRNA Single Cell LP	Takara Bio USA	634788
Unique Dual Index Kit (289–384)	Takara Bio USA	634755
Unique Dual Index Kit (193–288)	Takara Bio USA	634754
Unique Dual Index (97–192)	Takara Bio USA	634753
Unique Dual Index (1–96)	Takara Bio USA	634752
Deposited data		
Neuron Sequencing Data	Hand-picked neuron	https://painseq.shinyapps.io/TG_Corneal_neurons/
All RNAseq Data	Hand-picked neurons and sorted TG/Corneal Immune cells	https://doi.org/10.5281/zenodo.17364394
Experimental models: Organisms/strains		
B6;129S-Gt(ROSA)26Sortm32(CAG-COP4*H134R/EYFP)Hze/J	Jackson Laboratories	RRID:IMSR_JAX:012569
B6.129-Trpv1tm1(cre)Bbm/J	Jackson Laboratories	RRID:IMSR_JAX:017769
B6.Cg-Ccl2tm1.1Pame/J	Jackson Laboratories	RRID:IMSR_JAX:016849
C57BL/6 mice	Jackson Laboratories	RRID:IMSR_JAX:000664
Software and algorithms		
Seurat v4.3.0	10X Genomics	Nat. Comms 2017: 8:14049; Nat. Biotech, 2019, 37:925
Imaris Neuroscientist Package	Oxford Instruments	https://imaris.oxinst.com/products/imaris-for-neuroscientists
FV10-AS2 2.0	Evident Scientific (Olympus)	https://evidentscientific.com/en/downloads
Illumina DRAGEN FASTQ Generation	Illumina	https://www.illumina.com/products/by-type/informatics-products/basespace-sequence-hub/apps/dragen-fastq-toolkit.html
FlowJo v.10.10	BD Pharmingen	https://flowjo.com/flowjo10/download
Cell Ranger V.6.0		
CellChat		

## Highlights

- Cornea-innervating neurons, TG, and cornea immune cells were scRNA sequenced
- CellChat analysis and AI were used to predict neuron-immune interactions
- HSV-1 induces neuronal CCL2 to recruit myeloid cells to limit initial viral replication

## References

[1] Lu YZ, Nayer B, Singh SK, Alshoubaki YK, Yuan E, Park AJ, Maruyama K, Akira S, and Martino MM (2024). CGRP sensory neurons promote tissue healing via neutrophils and macrophages. Nature 628, 604–611. 10.1038/s41586-024-07237-y. 38538784 PMC11023938

[2] Balood M, Ahmadi M, Eichwald T, Ahmadi A, Majdoubi A, Roversi K, Roversi K, Lucido CT, Restaino AC, Huang S, (2022). Nociceptor neurons affect cancer immunosurveillance. Nature 611, 405–412. 10.1038/s41586-022-05374-w. 36323780 PMC9646485

[3] Ho TW, Edvinsson L, and Goadsby PJ (2010). CGRP and its receptors provide new insights into migraine pathophysiology. Nat. Rev. Neurol 6, 573–582. 10.1038/nrneurol.2010.127. 20820195

[4] Cohen JA, Edwards TN, Liu AW, Hirai T, Jones MR, Wu J, Li Y, Zhang S, Ho J, Davis BM, (2019). Cutaneous TRPV1(+) Neurons Trigger Protective Innate Type 17 Anticipatory Immunity. Cell 178, 919–932.e14. 10.1016/j.cell.2019.06.022. 31353219 PMC6788801

[5] Zhang S, Edwards TN, Chaudhri VK, Wu J, Cohen JA, Hirai T, Rittenhouse N, Schmitz EG, Zhou PY, McNeil BD, (2021). Nonpeptidergic neurons suppress mast cells via glutamate to maintain skin homeostasis. Cell 184, 2151–2166.e16. 10.1016/j.cell.2021.03.002. 33765440 PMC8052305

[6] Meerschaert KA, Edwards BS, Epouhe AY, Jefferson B, Friedman R, Babyok OL, Moy JK, Kehinde F, Liu C, Workman CJ, (2022). Neuronally expressed PDL1, not PD1, suppresses acute nociception. Brain Behav. Immun 106, 233–246. 10.1016/j.bbi.2022.09.001. 36089217 PMC10343937

[7] Cortez MA, Ivan C, Valdecanas D, Wang X, Peltier HJ, Ye Y, Araujo L, Carbone DP, Shilo K, Giri DK, (2016). PDL1 Regulation by p53 via miR-34. J. Natl. Cancer Inst 108, djv303. 10.1093/jnci/djv303. 26577528 PMC4862407

[8] Meerschaert KA, Adelman PC, Friedman RL, Albers KM, Koerber HR, and Davis BM (2020). Unique Molecular Characteristics of Visceral Afferents Arising from Different Levels of the Neuraxis: Location of Afferent Somata Predicts Function and Stimulus Detection Modalities. J. Neurosci 40, 7216–7228. 10.1523/JNEUROSCI.1426-20.2020. 32817244 PMC7534907

[9] Acosta MC, Belmonte C, and Gallar J (2001). Sensory experiences in humans and single-unit activity in cats evoked by polymodal stimulation of the cornea. J. Physiol 534, 511–525. 10.1111/j.1469-7793.2001.t01-1-00511.x. 11454968 PMC2278705

[10] Belmonte C, Acosta MC, and Gallar J (2004). Neural basis of sensation in intact and injured corneas. Exp. Eye Res 78, 513–525. 10.1016/j.exer.2003.09.023. 15106930

[11] Gonzalez-Gonzalez O, Bech F, Gallar J, Merayo-Lloves J, and Belmonte C (2017). Functional Properties of Sensory Nerve Terminals of the Mouse Cornea. Investig. Ophthalmol. Vis. Sci 58, 404–415. 10.1167/iovs.16-20033. 28118665

[12] Pina R, Ugarte G, Campos M, Inigo-Portugues A, Olivares E, Orio P, Belmonte C, Bacigalupo J, and Madrid R (2019). Role of TRPM8 Channels in Altered Cold Sensitivity of Corneal Primary Sensory Neurons Induced by Axonal Damage. J. Neurosci 39, 8177–8192. 10.1523/JNEUROSCI.0654-19.2019. 31471469 PMC6786815

[13] Murata Y, and Masuko S (2006). Peripheral and central distribution of TRPV1, substance P and CGRP of rat corneal neurons. Brain Res. 1085, 87–94. 10.1016/j.brainres.2006.02.035. 16564032

[14] Nakamura A, Hayakawa T, Kuwahara S, Maeda S, Tanaka K, Seki M, and Mimura O (2007). Morphological and immunohistochemical characterization of the trigeminal ganglion neurons innervating the cornea and upper eyelid of the rat. J. Chem. Neuroanat 34, 95–101. 10.1016/j.jchemneu.2007.05.005. 17587545

[15] Sasaoka A, Ishimoto I, Kuwayama Y, Sakiyama T, Manabe R, Shiosaka S, Inagaki S, and Tohyama M (1984). Overall distribution of substance P nerves in the rat cornea and their three-dimensional profiles. Investig. Ophthalmol. Vis. Sci 25, 351–356. 6199322

[16] Schecterson LC, Pazevic AA, Yang R, Matulef K, and Gordon SE (2020). TRPV1, TRPA1, and TRPM8 are expressed in axon terminals in the cornea: TRPV1 axons contain CGRP and secretogranin II; TRPA1 axons contain secretogranin 3. Mol. Vis 26, 576–587. 32863706 PMC7438417

[17] Nguyen MQ, Wu Y, Bonilla LS, von Buchholtz LJ, and Ryba NJP (2017). Diversity amongst trigeminal neurons revealed by high throughput single cell sequencing. PLoS One 12, e0185543. 10.1371/journal.pone.0185543. 28957441 PMC5619795

[18] Sharma N, Flaherty K, Lezgiyeva K, Wagner DE, Klein AM, and Ginty DD (2020). The emergence of transcriptional identity in somatosensory neurons. Nature 577, 392–398. 10.1038/s41586-019-1900-1. 31915380 PMC7307422

[19] Yang L, Xu M, Bhuiyan SA, Li J, Zhao J, Cohrs RJ, Susterich JT, Signorelli S, Green U, Stone JR, (2022). Human and mouse trigeminal ganglia cell atlas implicates multiple cell types in migraine. Neuron 110, 1806–1821.e8. 10.1016/j.neuron.2022.03.003. 35349784 PMC9338779

[20] Launay PS, Godefroy D, Khabou H, Rostene W, Sahel JA, Baudouin C, Melik Parsadaniantz S, and Reaux-Le Goazigo A (2015). Combined 3DISCO clearing method, retrograde tracer and ultramicroscopy to map corneal neurons in a whole adult mouse trigeminal ganglion. Exp. Eye Res 139, 136–143. 10.1016/j.exer.2015.06.008. 26072022

[21] Guerrero-Moreno A, Baudouin C, Melik Parsadaniantz S, and Réaux-Le Goazigo A (2020). Morphological and Functional Changes of Corneal Nerves and Their Contribution to Peripheral and Central Sensory Abnormalities. Front. Cell. Neurosci 14, 610342. 10.3389/fncel.2020.610342. 33362474 PMC7758484

[22] Acosta MC, Alfaro ML, Borrás F, Belmonte C, and Gallar J (2006). Influence of age, gender and iris color on mechanical and chemical sensitivity of the cornea and conjunctiva. Exp. Eye Res 83, 932–938. 10.1016/j.exer.2006.04.018. 16784741

[23] Khezri F, Mirzajani A, Karimian F, and Jafarzadehpur E (2015). Is Corneal Sensitivity Sex Dependent? J. Ophthalmic Vis. Res 10, 102–105. 10.4103/2008-322X.163772. 26425309 PMC4568604

[24] Caterina MJ, Leffler A, Malmberg AB, Martin WJ, Trafton J, Petersen-Zeitz KR, Koltzenburg M, Basbaum AI, and Julius D (2000). Impaired nociception and pain sensation in mice lacking the capsaicin receptor. Science 288, 306–313. 10.1126/science.288.5464.306. 10764638

[25] Woodbury CJ, Zwick M, Wang S, Lawson JJ, Caterina MJ, Koltzenburg M, Albers KM, Koerber HR, and Davis BM (2004). Nociceptors lacking TRPV1 and TRPV2 have normal heat responses. J. Neurosci 24, 6410–6415. 10.1523/JNEUROSCI.1421-04.2004. 15254097 PMC6729548

[26] Corey DP (2003). New TRP channels in hearing and mechanosensation. Neuron 39, 585–588. 10.1016/s0896-6273(03)00505-1. 12925273

[27] Bandell M, Story GM, Hwang SW, Viswanath V, Eid SR, Petrus MJ, Earley TJ, and Patapoutian A (2004). Noxious cold ion channel TRPA1 is activated by pungent compounds and bradykinin. Neuron 41, 849–857. 10.1016/s0896-6273(04)00150-3. 15046718

[28] Elitt CM, McIlwrath SL, Lawson JJ, Malin SA, Molliver DC, Cornuet PK, Koerber HR, Davis BM, and Albers KM (2006). Artemin overexpression in skin enhances expression of TRPV1 and TRPA1 in cutaneous sensory neurons and leads to behavioral sensitivity to heat and cold. J. Neurosci 26, 8578–8587. 10.1523/JNEUROSCI.2185-06.2006. 16914684 PMC6674358

[29] McMahon SB, and Wood JN (2006). Increasingly irritable and close to tears: TRPA1 in inflammatory pain. Cell 124, 1123–1125. 10.1016/j.cell.2006.03.006. 16564004

[30] Brierley SM, Hughes PA, Page AJ, Kwan KY, Martin CM, O’Donnell TA, Cooper NJ, Harrington AM, Adam B, Liebregts T, (2009). The ion channel TRPA1 is required for normal mechanosensation and is modulated by algesic stimuli. Gastroenterology 137, 2084–2095.e3. 10.1053/j.gastro.2009.07.048. 19632231 PMC2789877

[31] Kwan KY, Glazer JM, Corey DP, Rice FL, and Stucky CL (2009). TRPA1 modulates mechanotransduction in cutaneous sensory neurons. J. Neurosci 29, 4808–4819. 10.1523/JNEUROSCI.5380-08.2009. 19369549 PMC2744291

[32] Adelman PC, Baumbauer KM, Friedman R, Shah M, Wright M, Young E, Jankowski MP, Albers KM, and Koerber HR (2019). Single-cell q-PCR derived expression profiles of identified sensory neurons. Mol. Pain 15, 1744806919884496. 10.1177/1744806919884496. 31588843 PMC6820183

[33] Coste B, Mathur J, Schmidt M, Earley TJ, Ranade S, Petrus MJ, Dubin AE, and Patapoutian A (2010). Piezo1 and Piezo2 are essential components of distinct mechanically activated cation channels. Science 330, 55–60. 10.1126/science.1193270. 20813920 PMC3062430

[34] Coste B, Xiao B, Santos JS, Syeda R, Grandl J, Spencer KS, Kim SE, Schmidt M, Mathur J, Dubin AE, (2012). Piezo proteins are pore-forming subunits of mechanically activated channels. Nature 483, 176–181. 10.1038/nature10812. 22343900 PMC3297710

[35] Woo SH, Ranade S, Weyer AD, Dubin AE, Baba Y, Qiu Z, Petrus M, Miyamoto T, Reddy K, Lumpkin EA, (2014). Piezo2 is required for Merkel-cell mechanotransduction. Nature 509, 622–626. 10.1038/nature13251. 24717433 PMC4039622

[36] Hoffman PN, and Lasek RJ (1975). The slow component of axonal transport. Identification of major structural polypeptides of the axon and their generality among mammalian neurons. J. Cell Biol 66, 351–366. 10.1083/jcb.66.2.351. 49355 PMC2109569

[37] Liem RK, Yen SH, Salomon GD, and Shelanski ML (1978). Intermediate filaments in nervous tissues. J. Cell Biol 79, 637–645. 10.1083/jcb.79.3.637. 83322 PMC2110269

[38] Bennett GS, Tapscott SJ, DiLullo C, and Holtzer H (1984). Differential binding of antibodies against the neurofilament triplet proteins in different avian neurons. Brain Res. 304, 291–302. 10.1016/0006-8993(84)90333-0. 6430468

[39] Lawson SN, Harper AA, Harper EI, Garson JA, and Anderton BH (1984). A monoclonal antibody against neurofilament protein specifically labels a subpopulation of rat sensory neurones. J. Comp. Neurol 228, 263–272. 10.1002/cne.902280211. 6434599

[40] Lawson SN, and Waddell PJ (1991). Soma neurofilament immunoreactivity is related to cell size and fibre conduction velocity in rat primary sensory neurons. J. Physiol 435, 41–63. 10.1113/jphysiol.1991.sp018497. 1770443 PMC1181449

[41] Felipe CD, Gonzalez GG, Gallar J, and Belmonte C (1999). Quantification and immunocytochemical characteristics of trigeminal ganglion neurons projecting to the cornea: effect of corneal wounding. Eur. J. Pain 3, 31–39. 10.1053/eujp.1998.0100. 10700335

[42] McKemy DD, Neuhausser WM, and Julius D (2002). Identification of a cold receptor reveals a general role for TRP channels in thermosensation. Nature 416, 52–58. 10.1038/nature719. 11882888

[43] Nealen ML, Gold MS, Thut PD, and Caterina MJ (2003). TRPM8 mRNA is expressed in a subset of cold-responsive trigeminal neurons from rat. J. Neurophysiol 90, 515–520. 10.1152/jn.00843.2002. 12634279

[44] McKemy DD (2005). How cold is it? TRPM8 and TRPA1 in the molecular logic of cold sensation. Mol. Pain 1, 16. 10.1186/1744-8069-1-16. 15847696 PMC1087877

[45] Bhuiyan SA, Xu M, Yang L, Semizoglou E, Bhatia P, Pantaleo KI, Tochitsky I, Jain A, Erdogan B, Blair S, (2024). Harmonized cross-species cell atlases of trigeminal and dorsal root ganglia. Sci. Adv 10, eadj9173. 10.1126/sciadv.adj9173. 38905344 PMC11804847

[46] Huang S, Ziegler CGK, Austin J, Mannoun N, Vukovic M, Ordovas-Montanes J, Shalek AK, and von Andrian UH (2021). Lymph nodes are innervated by a unique population of sensory neurons with immune-modulatory potential. Cell 184, 441–459.e25. 10.1016/j.cell.2020.11.028. 33333021 PMC9612289

[47] Jain A, Gyori BM, Hakim S, Jain A, Sun L, Petrova V, Bhuiyan SA, Zhen S, Wang Q, Kawaguchi R, (2024). Nociceptor-immune interactomes reveal insult-specific immune signatures of pain. Nat. Immunol 25, 1296–1305. 10.1038/s41590-024-01857-2. 38806708 PMC11224023

[48] Kwon MJ, Shin HY, Cui Y, Kim H, Thi AHL, Choi JY, Kim EY, Hwang DH, and Kim BG (2015). CCL2 Mediates Neuron-Macrophage Interactions to Drive Proregenerative Macrophage Activation Following Preconditioning Injury. J. Neurosci 35, 15934–15947. 10.1523/JNEUROSCI.1924-15.2015. 26631474 PMC6605453

[49] Wu M, Hill LJ, Downie LE, and Chinnery HR (2022). Neuroimmune crosstalk in the cornea: The role of immune cells in corneal nerve maintenance during homeostasis and inflammation. Prog. Retin. Eye Res 91, 101105. 10.1016/j.preteyeres.2022.101105. 35868985

[50] Wang B, Jiang B, Li GW, Dong F, Luo Z, Cai B, Wei M, Huang J, Wang K, Feng X, (2023). Somatosensory neurons express specific sets of lincRNAs, and lincRNA CLAP promotes itch sensation in mice. EMBO Rep. 24, e54313. 10.15252/embr.202154313. 36524339 PMC9900349

[51] Flauaus C, Engel P, Zhou F, Petersen J, Ruth P, Lukowski R, Schmidtko A, and Lu R (2022). Slick Potassium Channels Control Pain and Itch in Distinct Populations of Sensory and Spinal Neurons in Mice. Anesthesiology 136, 802–822. 10.1097/ALN.0000000000004163. 35303056

[52] Jin S, Guerrero-Juarez CF, Zhang L, Chang I, Ramos R, Kuan CH, Myung P, Plikus MV, and Nie Q (2021). Inference and analysis of cell-cell communication using CellChat. Nat. Commun 12, 1088. 10.1038/s41467-021-21246-9. 33597522 PMC7889871

[53] Epouhe A, Jones MR, Najjar SA, Cohen JA, Kaplan DH, Koerber HR, and Albers KM (2022). Use of OptogeneticsOptogenetics for the Study of Skin–Nerve Communication. In Contemporary Approaches to the Study of Pain: From Molecules to Neural Networks, Seal RP, ed. (Springer US), pp. 333–346. 10.1007/978-1-0716-2039-7_17.

[54] Yin XT, Hartman A, Sirajuddin N, Shukla D, Leger AS, Keadle TL, and Stuart PM (2024). UVB induced reactivation leads to HSV1 in the corneas of virtually all latently infected mice and requires STING to develop corneal disease. Sci. Rep 14, 6859. 10.1038/s41598-024-52597-0. 38514671 PMC10957950

[55] Conrady CD, Zheng M, Mandal NA, van Rooijen N, and Carr DJJ (2013). IFN-alpha-driven CCL2 production recruits inflammatory monocytes to infection site in mice. Mucosal Immunol. 6, 45–55. 10.1038/mi.2012.46. 22692455 PMC3449026

[56] Tumpey TM, Chen SH, Oakes JE, and Lausch RN (1996). Neutrophil-mediated suppression of virus replication after herpes simplex virus type 1 infection of the murine cornea. J. Virol 70, 898–904. 10.1128/JVI.70.2.898-904.1996. 8551629 PMC189893

[57] Yun H, Yee MB, Lathrop KL, Kinchington PR, Hendricks RL, and St Leger AJ (2020). Production of the Cytokine VEGF-A by CD4(+) T and Myeloid Cells Disrupts the Corneal Nerve Landscape and Promotes Herpes Stromal Keratitis. Immunity 53, 1050–1062.e5. 10.1016/j.immuni.2020.10.013. 33207210 PMC7682749

[58] Yun H, Lathrop KL, and St Leger AJ (2021). A whole-mount immunohistochemistry protocol for detection of mouse corneal nerves. STAR Protoc. 2, 100734. 10.1016/j.xpro.2021.100734. 34430909 PMC8371250

[59] Malin SA, Davis BM, and Molliver DC (2007). Production of dissociated sensory neuron cultures and considerations for their use in studying neuronal function and plasticity. Nat. Protoc 2, 152–160. 10.1038/nprot.2006.461. 17401349

[60] Bray NL, Pimentel H, Melsted P, and Pachter L (2016). Near-optimal probabilistic RNA-seq quantification. Nat. Biotechnol 34, 525–527. 10.1038/nbt.3519. 27043002

[61] Butler A, Hoffman P, Smibert P, Papalexi E, and Satija R (2018). Integrating single-cell transcriptomic data across different conditions, technologies, and species. Nat. Biotechnol 36, 411–420. 10.1038/nbt.4096. 29608179 PMC6700744

[62] Hafemeister C, and Satija R (2019). Normalization and variance stabilization of single-cell RNA-seq data using regularized negative binomial regression. Genome Biol. 20, 296. 10.1186/s13059-019-1874-1. 31870423 PMC6927181

