# Supplementary material for: The production of the chemokine CCL2 by corneal sensory neurons initiates anti-viral immunity at the cornea and trigeminal ganglion
Source: Cell Rep. Author manuscript; Available in PMC 2026 Apr 1. (PMC13042276; doi:10.1016/j.celrep.2025.116693)
Supplement: 1 [file NIHMS2142907-supplement-1.pdf]

Cell Reports, Volume *45*

Supplemental information

The production of the chemokine CCL2 by corneal sensory neurons initiates anti-viral immunity at the cornea and trigeminal ganglion

Hongmin Yun, Kaveh Moghbeli, Peter Habib Gerges, Rylee N. Cisney, Masaaki Yoshida, William F. Hawse, William A. MacDonald, Shamsuddin A. Bhuiyan, William Renthal, Christopher J. Sullivan, Jishnu Das, Daniel H. Kaplan, Harinder Singh, Brian M. Davis, and Anthony J. St. Leger

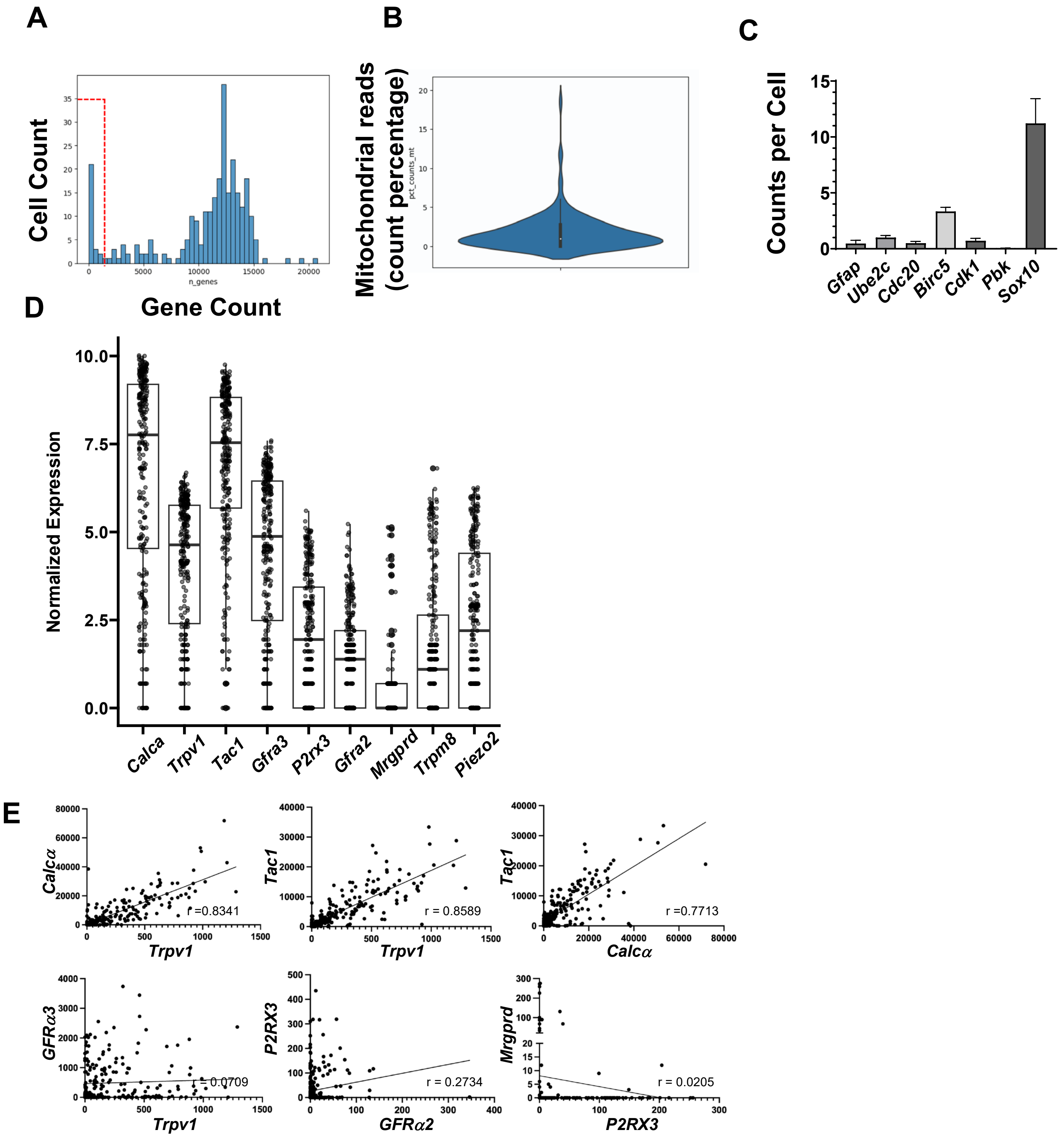

Supplemental Figure 1. Quality control of neurons scRNAseq transcripts. A) Total gene counts of individual, hand-picked neurons after scRNAseq analysis. The dotted red box contains cells that were omitted from analysis due to insufficient gene counts. The average gene counts per cells was 12,000. **B**) Mitochondrial read percentages of scRNAseq data from hand-picked neurons. Overall mitochondrial reads were less than 10%. **C**) Raw data counts for genes indicating glia cells were quantified. Bars represent the mean counts ± SEM for all sequenced neurons. **D**) Counts of RNA for individual neurons were quantified and normalized to housekeeping genes. The most highly expressed transcripts identified for peptidergic afferents (*Calca* and *Tac1)* and the lowest expressed transcripts were associated with non-peptidergic afferents (e.g., *Mrgprd* and *Gfra2*). **E**) Raw data counts for transcripts associated with peptidergic neurons (*Calca*,*Tac1, Trpv1,* but not *Gfra3*) and non-peptidergic neurons (*P2rx3, GFRα2, Mrgprd*) were plotted against each other to assess the correlations between genes.

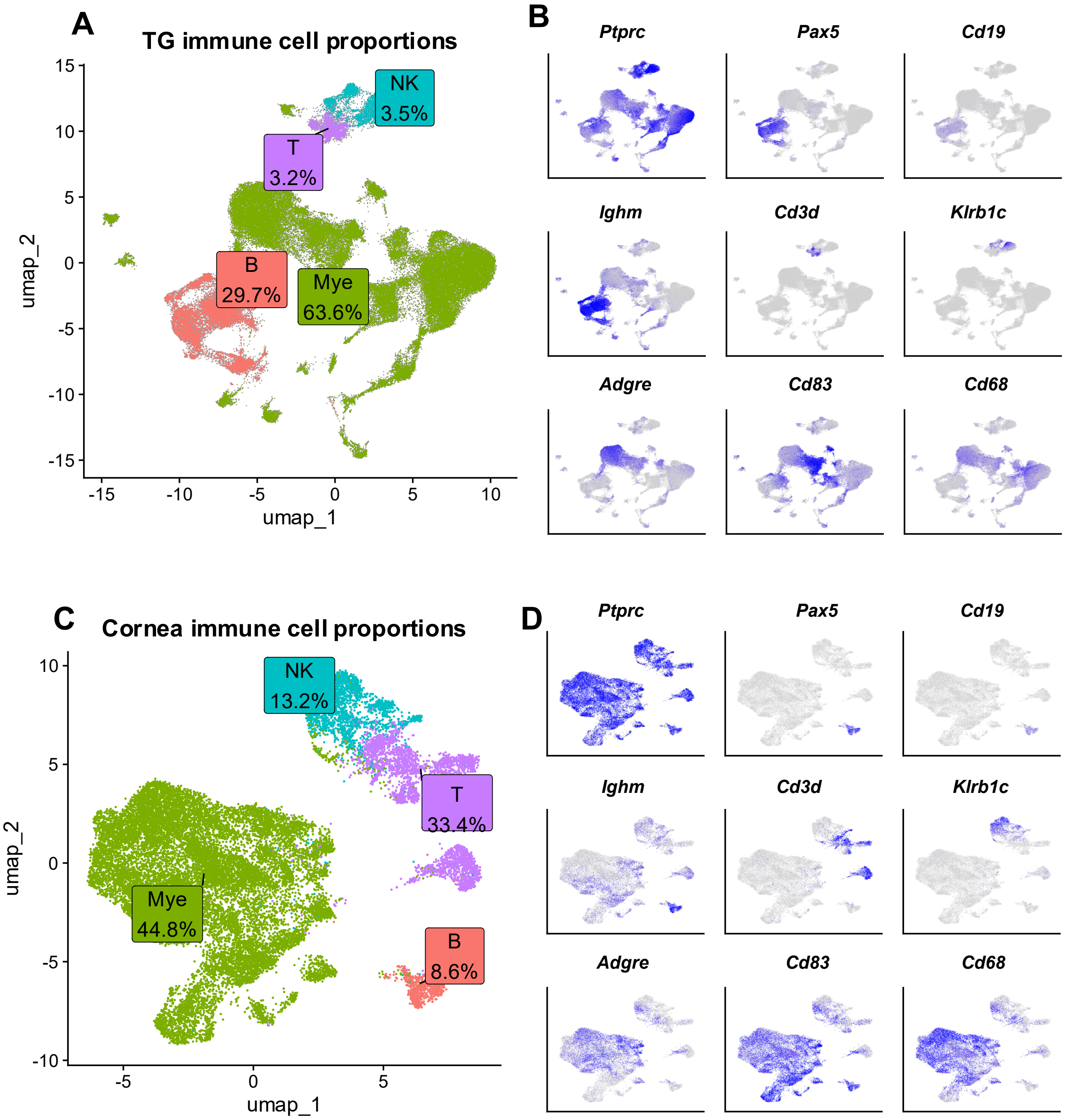

Supplemental Figure 2. Single-cell annotation of immune cells in the trigeminal ganglion and cornea. A) UMAP identifying the subsets of CD45+ immune cells and their relative proportions within the mouse trigeminal ganglion. **B**) Representative marker genes used for TG immune cell type identification. **C**) UMAP identifying the subsets of CD45+ immune cells and their relative proportions within the mouse cornea. **D**) Representative marker genes used for corneal immune cell type identification.

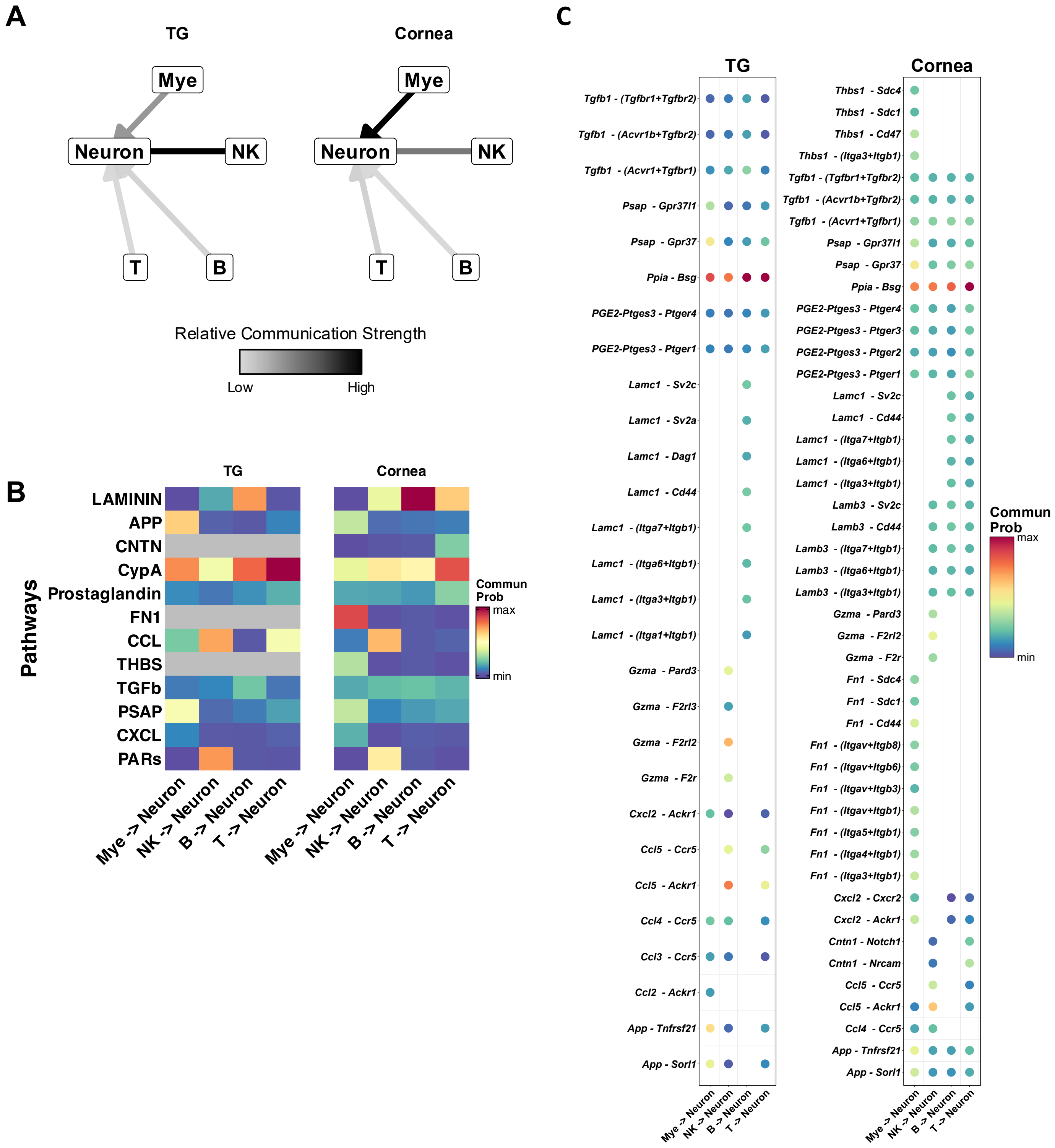

Supplemental Figure 3. Potential immune cell to neuron interactions in the mouse trigeminal ganglion and cornea. A) Relative communication strength for signaling from live immune cells (CD45$^+$Ly6G$^-$EpCam$^-$ cells) the TG and cornea to mouse corneal afferents. **B**) Relative communication probabilities (per anatomic location) for potential immune cell to neuron signaling pathways (grey color indicates no significant interaction detected). **C**) Relative communication probabilities of significant immune cell ligand and neuronal receptor pairs ($p < 0.05$ by permutation testing).

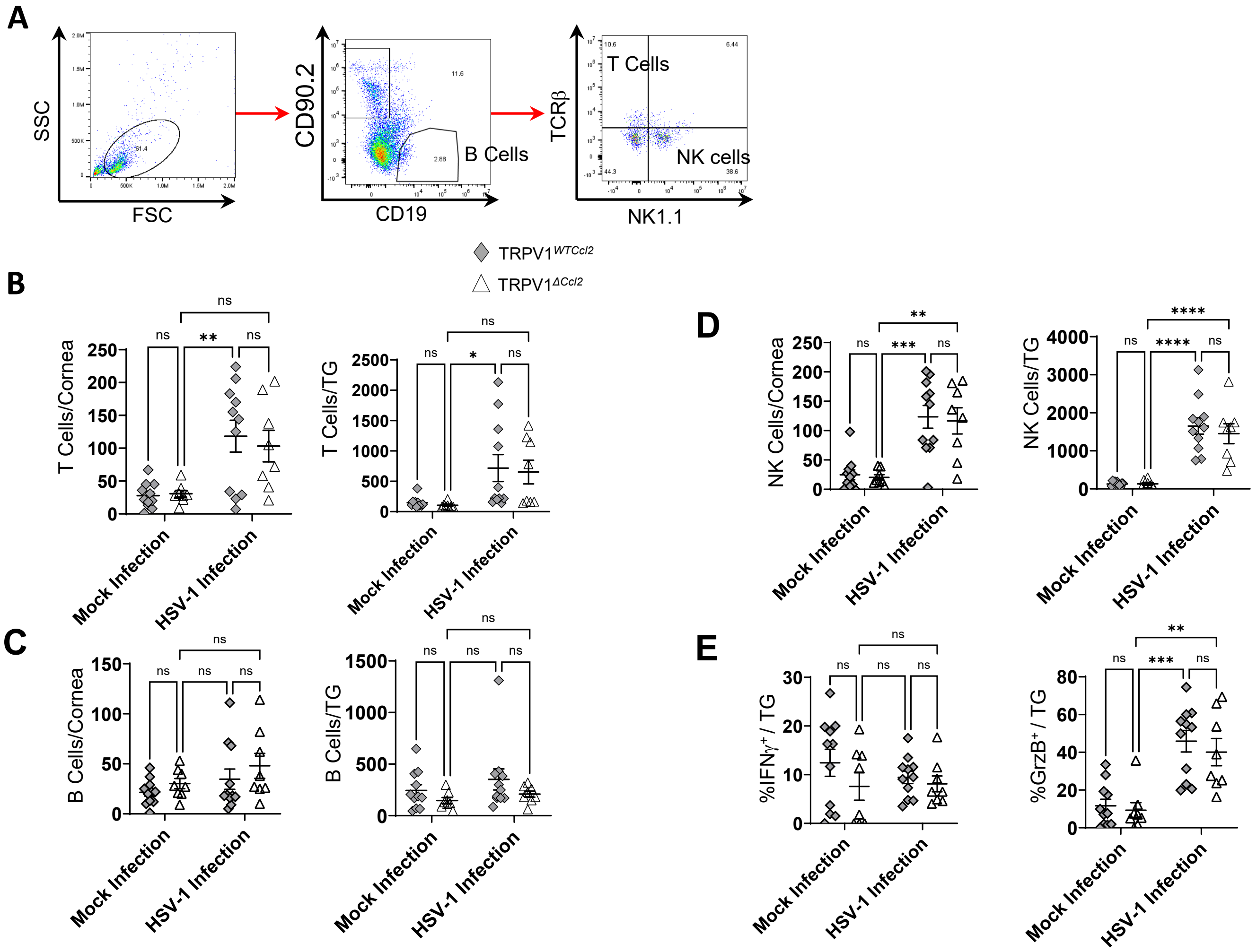

Supplemental Figure 4. Neuronal CCL2 production does not influence lymphocyte recruitment to the cornea or TG after HSV-1 infection. The eyes of TRPV1$^{WT\ Ccl2}$ or TRPV1$^{\Delta Ccl2}$ mice were scarified and infected in one eye with 1x10^5 PFU of HSV-1 (KOS Strain) or received PBS in the contralateral eye as in **Figure 4**[56]. On day 3 post infection, mice were sacrificed, and immune cells were analyzed by flow cytometry. (**A**) Representative flow plots illustrating gating strategy for T, B, and NK Cells in cornea and TG. (**B**) Quantification of TCRβ$^+$ cells in the cornea and TG. (**C**) Quantification of CD19$^+$ Cells in the cornea and TG. (**D**) Quantification of NK Cells in the cornea and TG. (**E**) Frequency of TG NK cells that were producing IFNγ and/or GrzB. Data were pooled from two independent experiments. Each symbol represents an individual mouse. Statistical significance was determined using a Two-Way ANOVA where all means from all groups were compared in each graph._These data confirm that deletion of CCL2 from neurons does not affect lymphocyte migration into the cornea. Expectedly, (F) NK cells outnumber (B) T cells in the TG and cornea given that NK predominate the acute recruitment of lymphocytes to both sites. Similarly, (C) B cells are not recruited in appreciable numbers at either tissue. We conclude from these data that lymphocytes are not recruited by CCL2 produced by neurons during acute HSV-1 infection. These cells are likely recruited either by: 1) other chemokines produced by neurons, or 2) cytokines/chemokines produced by epithelial cells/myeloid cells.

Supplemental TABLE LEGENDS

Table 1. Genes detected in single, hand-picked hand-picked corneal afferents.
Raw counts for Full-length single-cell RNA-seq library we prepared using full-length cDNA was generated with the Takara SMART-Seq Single-cell kit (Takara: 634473) according to the manufacturer's instructions. Libraries from each individually collected cell were pooled by animal (in equal volume) prior to library cleanup using Agencourt Ampure XP beads. Library pool quantification and assessment was done using a Qubit FLEX fluorometer and an Agilent Fragment Analyzer 5300. Libraries were normalized and pooled to 2nM by calculating the concentration based off the fragment size (base pairs) and the concentration (ng/μl) of the libraries. Sequencing was performed on an Illumina NextSeq 2000, using a P2 300 flow cell. The pooled library was loaded at 750 pM. Sequencing was carried out 2x151 bp, with a target of 1 million reads per sample. Sequencing data was demultiplexed by the on-board Illumina DRAGEN FASTQ Generation software (v3.10.12). Of the 284 samples, 254 produced high quality RNAseq libraries (ca. 88% of all samples submitted). Transcript abundance was performed with Kallisto (v0.46.1) using an index based off the Ensembl v96 reference transcriptome for mus musculus [57].

Table 2. Marker genes for corneal afferent clusters. Unsupervised Leiden clustering of single-cell transcriptomes from corneal afferents identified 10 groupings of peptidergic and non-peptidergic neurons. Differentially expressed genes for each cluster are presented. P-values obtained via Wilcoxon rank sum performed on each cluster compared to all remaining clusters and adjusted with the Benjamini-Hochberg false discovery correction.
